# Supplementary material for: Contrastive learning enables epitope overlap predictions for targeted antibody discovery
Source: Patterns (N Y). 2025 Nov 13;7(2):101419. doi: 10.1016/j.patter.2025.101419 (PMC12921510; doi:10.1016/j.patter.2025.101419)
Supplement: Document S1. Figures S1–S4 [file mmc1.pdf]

Patterns, Volume *7*

Supplemental information

Contrastive learning enables epitope overlap predictions for targeted antibody discovery

Clinton M. Holt, Alexis K. Janke, Parastoo Amlashi, Parker J. Jamieson, Toma M. Marinov, and Ivelin S. Georgiev

Patterns, Volume *7*

Supplemental information

Contrastive learning enables epitope overlap predictions for targeted antibody discovery

Clinton M. Holt, Alexis K. Janke, Parastoo Amlashi, Parker J. Jamieson, Toma M. Marinov, and Ivelin S. Georgiev

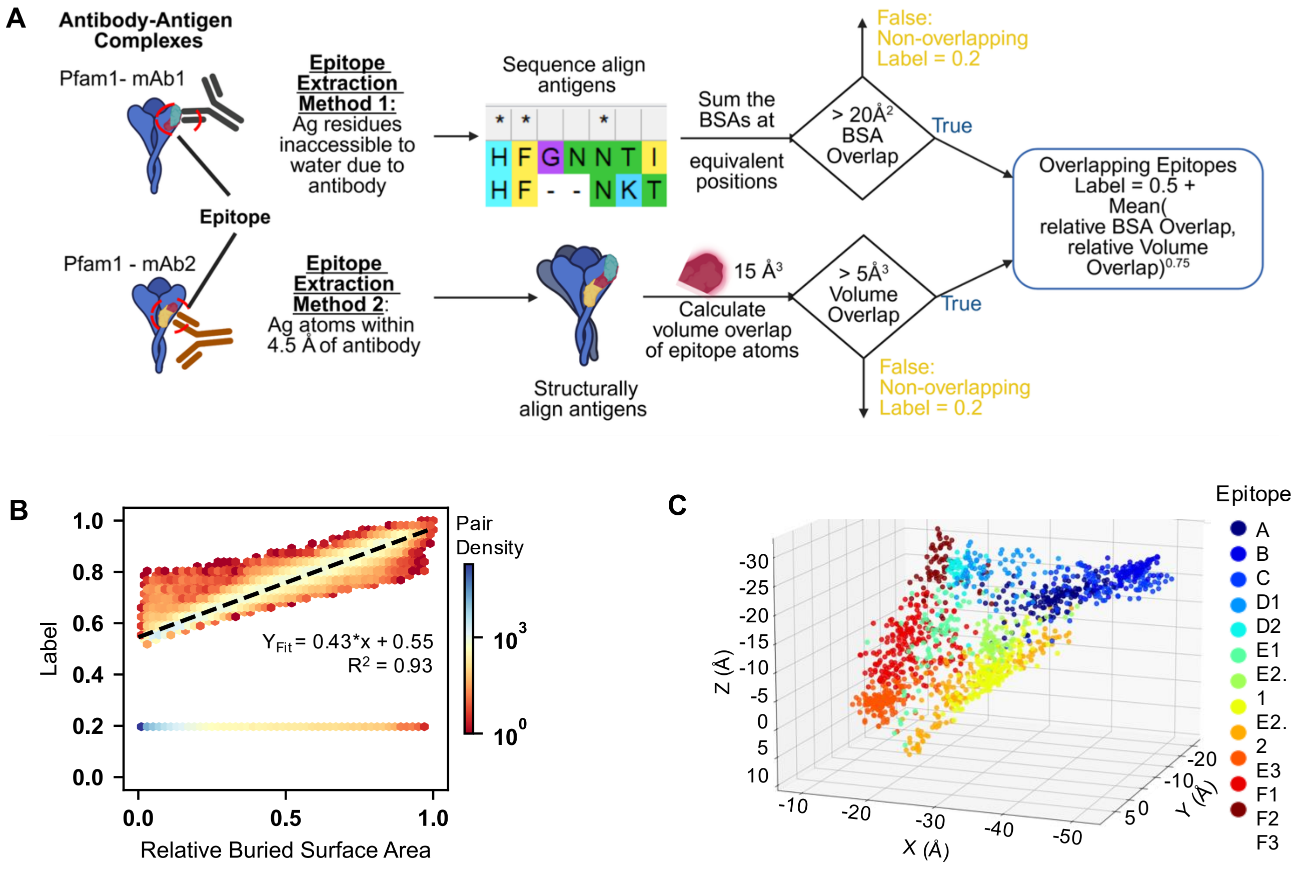

Figure S1. Quantitative Framework for Defining Epitope Relationships and Dataset Characteristics. (A) Approach for determining machine learning labels for antibody pairs targeting antigens within the same Pfam family. Two complementary metrics are employed. First, buried solvent accessible surface area (BSA) based residue-level epitope overlap, calculated as the solvent-accessible surface area buried by both antibodies at equivalent residues based on a pairwise sequence alignment. Antibody pairs where this value is less than 20 Å² are labeled as having non-overlapping epitopes. Second distance-based atom-level epitope overlap is calculated, defined by the volume overlap of antigen atoms within 4.5 Å of each antibody following antigen structural alignment (threshold >5 Å³). Finally, the epitope overlap is divided by the maximum possible epitope overlap to get relative overlaps. (B) Relationship between machine learning labels and relative buried surface area overlap visualized through hexagonal binning density plot. Only pairs with labels ≥ 0.5 were included for regression. Color intensity indicates pair density from minimum (red) to maximum (blue). (C) Three-dimensional visualization of deep mutational scanning (DMS) weighted average coordinates, with points colored by epitope classification. Coordinate calculation methodology is detailed in Methods, but briefly the antibody epitope is mapped onto the coordinates of SARS-CoV-2 RBD weighted by the extent each residue contributes to escape.

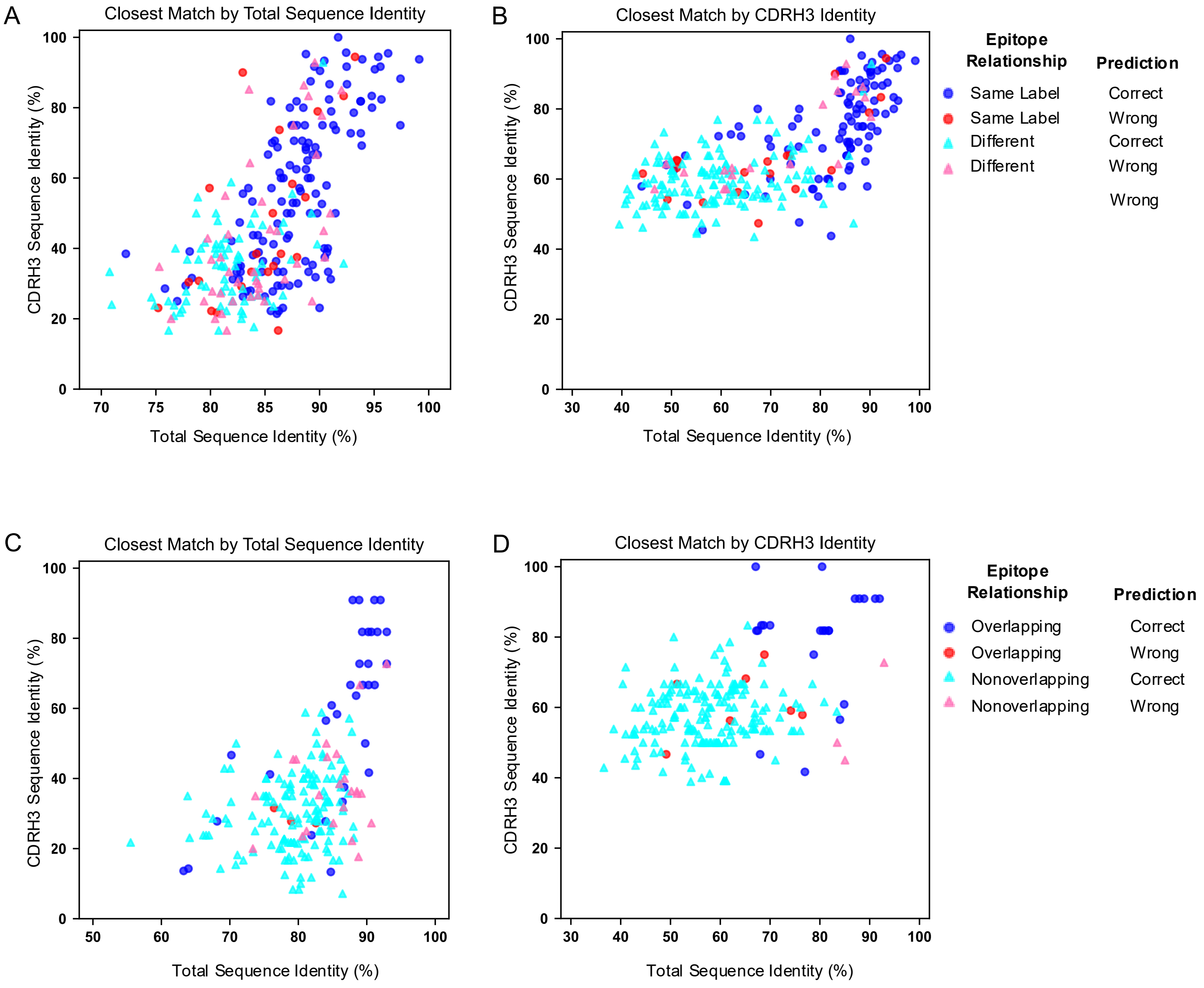

Figure S2: Test set antibodies have minimal sequence overlap with the training set. A-B) All SARS-CoV-2 RBD DMS test set antibodies are plotted as a single point representing how similar the most similar training set antibody is to it. The closest training set antibody is chosen by having the highest A) total percent amino acid sequence identity over both the heavy and light chains or B) CDRH3 percent amino acid sequence identity. C-D). The same analysis is repeated for the SAbDab test set antibodies. Pairings which have non-overlapping epitopes on the same pfam or bind different pfams are grouped together.

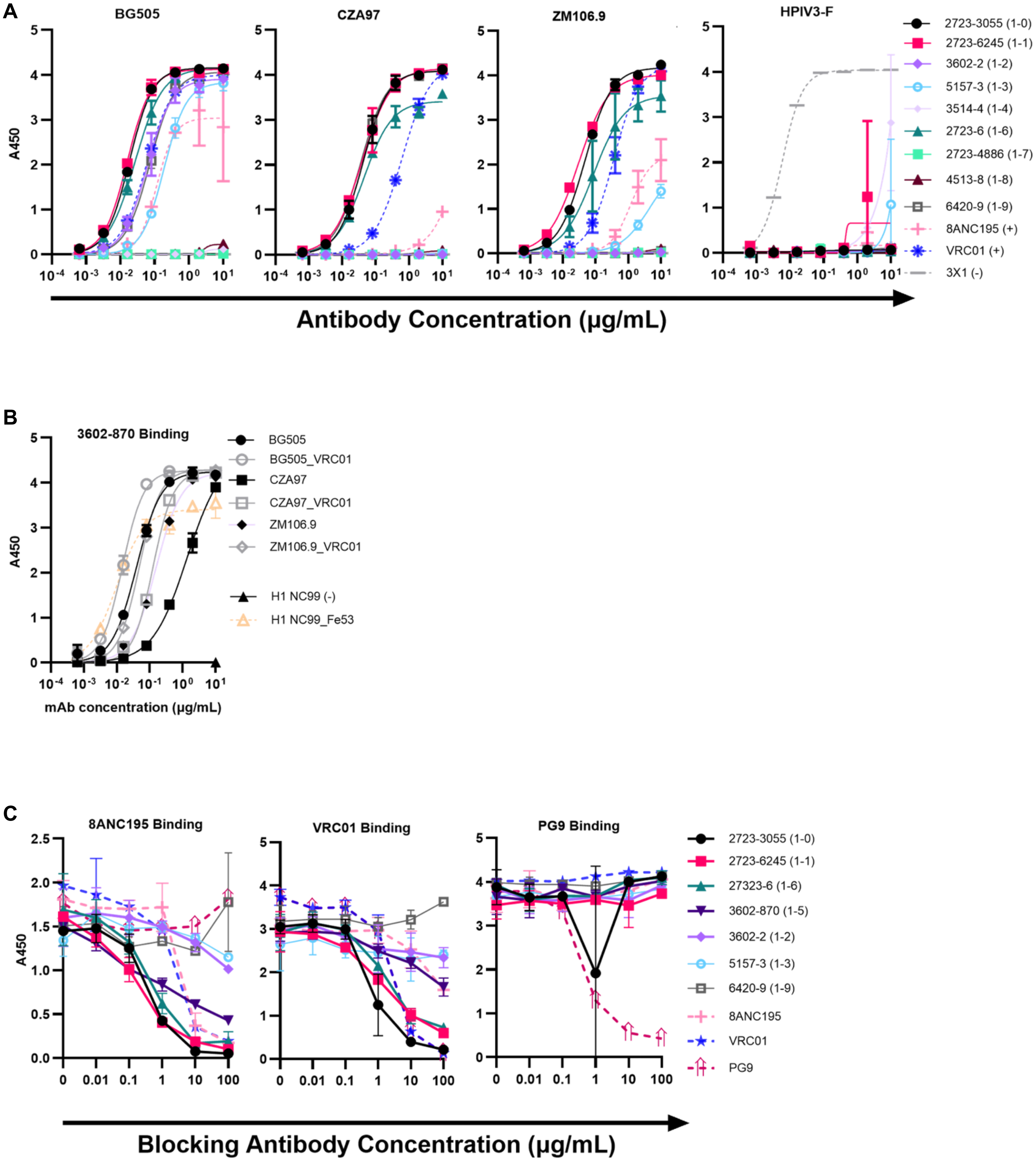

Figure S3: ELISA data for 8ANC195-targeted antibody discovery campaign. (A-B) ELISA curves pertaining to Figure 5C AUC values. Binding to trimeric HIV-1 envelope (BG505, CZA97, ZM106.9) or to a negative control antigen, human parainfluenza virus 3 fusion protein (HPIV3-F). (B) ELISA curves for antibody 3602-870, reproduced from data collected from 3602-870's initial publication. Antigens listed without underscores refer to the use of 3602-870 as the primary antibody whereas antibodies after underscores are positive controls for that antigen. H1 NC99 stands for the Influenza A hemagglutinin from the strain A/New Caledonia/20/99 (H1). (C) Competition ELISA curves showing detection of biotinylated antibody binding to BG505 SOSIP.664 with the biotinylated antibody as the title (8ANC195, VRC01, or PG9). Absorbance at 450 nm shown in presence of increasing concentrations of competitor antibodies. Data are represented as mean ± standard deviation. Filled symbols indicate mAbs displaying competition with 8ANC195.

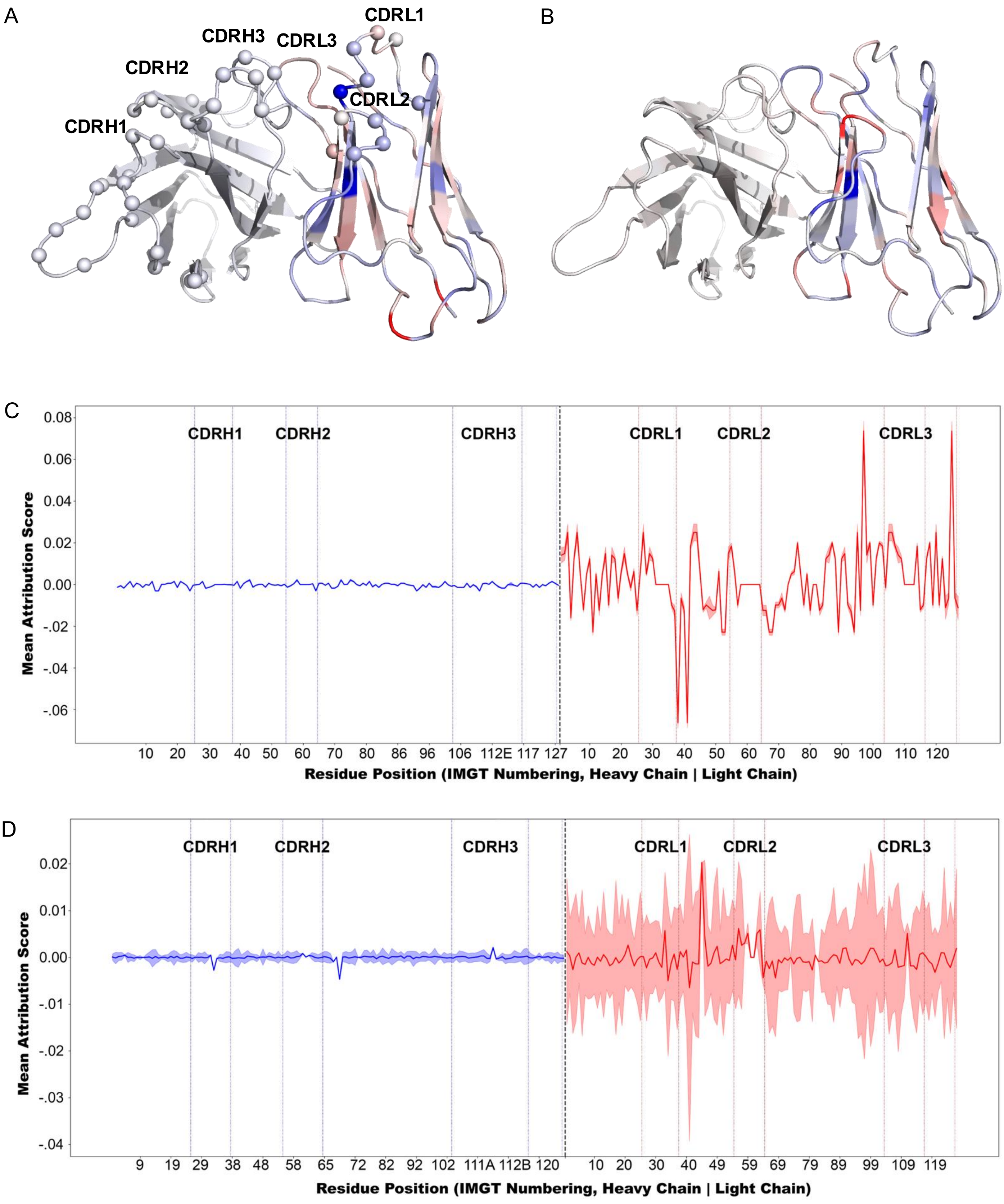

Figure S4. Feature Attribution Analysis Reveals the Light Chain Dominates Epitope Overlap Predictions. (A) Mean feature attributions for the 8ANC195 antibody (PDB ID 7KDE) versus five epitope-overlapping antibodies, mapped onto its structure. Complementarity-determining regions (CDRs) are labeled, and antigen-contacting residues (heavy atoms within 4.5 Å) are shown as spheres. (B) Mean feature attributions averaged over all test antibodies versus their nearest training neighbor, mapped onto the same structure for comparison. In both structures, colors scale from negative (blue) to positive (red) attribution. (C) The per-residue attribution scores used for panel A, plotted for the heavy (blue) and light (red) chains with CDRs highlighted. (D) The per-residue attribution scores used for panel B, averaged at each IMGT position across the test set; the shaded region represents one standard deviation.
