# Supplementary material for: Contrastive learning enables epitope overlap predictions for targeted antibody discovery
Source: Patterns (N Y). 2025 Nov 13;7(2):101419. doi: 10.1016/j.patter.2025.101419 (PMC12921510; doi:10.1016/j.patter.2025.101419)
Supplement: Document S2. Article plus supplemental information [file mmc2.pdf]

Patterns

Article

Contrastive learning enables epitope overlap predictions for targeted antibody discovery

Graphical abstract

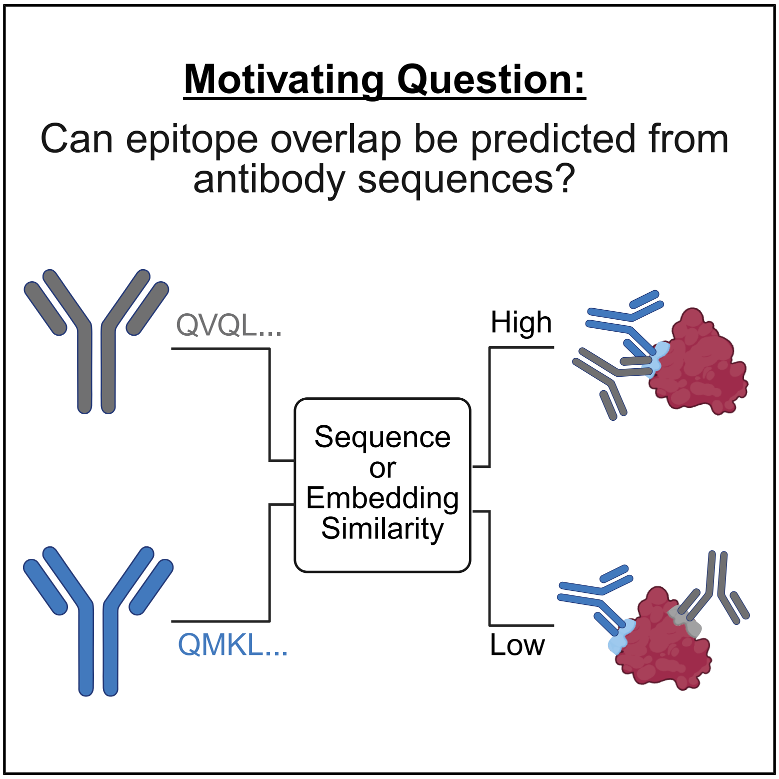

Authors

Clinton M. Holt, Alexis K. Janke, Parastoo Amlashi, Parker J. Jamieson, Toma M. Marinov, Ivelin S. Georgiev

Correspondence

ivelin.georgiev@vumc.org

In brief

Antibodies are important for human health, but their diversity makes computational prediction of their binding properties challenging. Through contrastive fine-tuning of antibody language models on millions of antibody pairs, the authors enable accurate prediction of epitope overlap even among sequence-diverse antibodies, providing powerful new tools for therapeutic antibody discovery.

Highlights

- Contrastive fine-tuning encodes epitope relationships into antibody LLM embeddings
- AbLang-PDB identifies overlapping-epitope antibodies across 250 protein families
- Both structural and binding data successfully teach epitope specificity
- AbLang-PDB achieved a 50% hit rate, identifying 8ANC195 overlapping-epitope antibodies

Holt et al., 2026, Patterns 7, 101419
February 13, 2026 © 2025 The Author(s). Published by Elsevier Inc.
https://doi.org/10.1016/j.patter.2025.101419

CellPress

Patterns
CellPress OPEN ACCESS

Article

Contrastive learning enables epitope overlap predictions for targeted antibody discovery

Clinton M. Holt,[1,2,3] Alexis K. Janke,[1,4] Parastoo Amlashi,[1,4] Parker J. Jamieson,[1,4] Toma M. Marinov,[1,3,4] and Ivelin S. Georgiev[1,2,3,4,5,6,7,8,9,10,11,*]
[1]Vanderbilt Center for Antibody Therapeutics, Vanderbilt University Medical Center, Nashville, TN 37232, USA
[2]Program in Chemical and Physical Biology, Vanderbilt University Medical Center, Nashville, TN 37232, USA
[3]Center for Computational Microbiology and Immunology, Vanderbilt University Medical Center, Nashville, TN 37232, USA
[4]Department of Pathology, Microbiology, and Immunology, Vanderbilt University Medical Center, Nashville, TN 37232, USA
[5]Department of Computer Science, Vanderbilt University, Nashville, TN 37232, USA
[6]Department of Biomedical Informatics, Vanderbilt University, Nashville, TN 37232, USA
[7]Department of Chemical and Biomolecular Engineering, Vanderbilt University, Nashville, TN 37232, USA
[8]Department of Biochemistry, Vanderbilt University, Nashville, TN 37232, USA
[9]Vanderbilt Institute for Infection, Immunology, and Inflammation, Vanderbilt University Medical Center, Nashville, TN 37232, USA
[10]Center for Structural Biology, Vanderbilt University, Nashville, TN 37232, USA
[11]Lead contact
*Correspondence: ivelin.georgiev@vumc.org
https://doi.org/10.1016/j.patter.2025.101419

THE BIGGER PICTURE Developing vaccines and antibody drugs requires identifying where antibodies bind to target proteins. Current computational methods face a fundamental trade-off: simple sequence comparisons are reliable when applicable but miss most promising candidates, while complex structural approaches have broader applicability but require significant computational resources and often produce inaccurate predictions. This bottleneck significantly slows therapeutic development, particularly for challenging targets such as rapidly mutating viruses. We developed a machine learning approach that teaches antibody language models to recognize when different antibodies will bind to overlapping antigen sites, even when the antibody sequences are significantly different. Our approach offers a practical solution for rapidly screening large antibody databases, potentially accelerating the discovery pipeline by identifying the most promising candidates before expensive laboratory validation.

SUMMARY

Computational epitope prediction remains an unmet need for therapeutic antibody development. We present three complementary approaches for predicting epitope relationships from antibody sequences. First, by analyzing approximately 18 million antibody pairs targeting around 250 protein families, we establish that over 70% of heavy-chain complementarity-determining region 3 (CDRH3) sequence identity among antibodies sharing both V genes reliably predicts overlapping epitopes. Second, we develop a supervised contrastive fine-tuning framework for antibody large language models that enriches embeddings with epitope information. Applied to SARS-CoV-2 receptor-binding-domain antibodies, this approach achieves 97% total accuracy in predicting high levels of structural overlap. Third, we create AbLang-PDB, a generalized model achieving 5-fold improvement in average precision over sequence-based methods and correlating strongly with epitope overlap ($\rho = 0.81$). Experimental validation with HIV-1 antibody 8ANC195 shows that 70% of selected candidates demonstrate HIV-1 specificity and 50% compete for binding. These models provide powerful tools for epitope-targeted antibody discovery while demonstrating contrastive learning's efficacy for encoding epitope information.

INTRODUCTION

Monoclonal antibody (mAb) therapeutics have revolutionized modern medicine since their first US Food and Drug Administration (FDA) approval in 1986, with blockbuster treatments for cancers, autoimmune diseases, and infectious diseases generating billions in annual revenue.[1] Beyond therapeutics, antibodies serve as fundamental research tools and provide crucial insights

© 2025 The Author(s). Published by Elsevier Inc.
This is an open access article under the CC BY license (http://creativecommons.org/licenses/by/4.0/).

into immune responses to vaccines and pathogens. Despite their clinical success, developing therapeutic antibodies remains resource intensive, with epitope characterization—identifying the specific region on an antigen where an antibody binds—posing a significant bottleneck.[2] For example, in the development of broadly neutralizing antibodies (bNAbs) against HIV-1, epitope mapping is critical to ensuring efficacy across diverse viral strains.[3]

Epitope characterization typically proceeds through three complementary approaches: (1) structural mapping to define physical contact points between antibody and antigen, (2) functional mapping to identify binding-critical residues through mutation, and (3) competition binding experiments to group antibodies that interfere with each other's binding. Each approach helps guide therapeutic development, whether identifying sites of vulnerability on pathogens or developing complementary antibody combinations.[4–6]

Understanding the similarities and differences (or the level of overlap) between the epitopes of different antibody candidates provides critical information that can be utilized when developing antibody therapeutics. For example, in pandemic response efforts against a newly emerging virus, the selection of two or more non-competing antibodies that synergize to form a more effective drug than either individual antibody can be critical for counteracting potential virus escape. In other cases, identifying multiple antibodies against the same functionally important epitope can provide a larger set of candidates for further evaluation, down-selection, and development.

While experimental approaches for antibody epitope characterization are undoubtedly effective, computational approaches can present an efficient and cost-effective alternative. Generally, computational approaches can interrogate the relationship between antibody sequence features and epitope similarity in order to predict the level of epitope overlap between antibody candidates. These approaches range from direct comparisons of the full amino acid sequence or just the complementarity-determining region 3 (CDR3) amino acid sequence within gene groups to comparing predicted structures or predicted antigen-binding residues.[5,7–16] While the direct sequence-based methods have shown success in clustering functionally related antibodies, the antibody sequence similarity thresholds utilized by these approaches have been rigorously validated for only a few antigens and epitopes.[5,8–10,17] The indirect approaches allow for searching a broader antibody sequence space, but levels of accuracy are low and unable to detect overlapping-epitope antibodies using distinct structural mechanisms, such as targeting the same site from different angles—an aspect that can significantly influence Fc effector functions and binding breadth.[16,18–20] This limitation is particularly problematic when searching for therapeutic candidates, where expanding the candidate pool beyond highly similar structures could be necessary to overcome challenges such as low yields or suboptimal binding properties.[21]

To overcome the limitations of simple sequence identity thresholds, a variety of computational tools have been developed, ranging from structure-based models that predict antigen-binding residues[22–24] to general protein language models, such as ESM-2,[25] to antibody-specific language models, including Parapred,[26] AntiBERTy,[27] AbLang,[28] AbLang2,[29] IgBert,[30] and BALM.[31] Among these, AbLang has emerged as particularly influential, having been trained on millions of naturally occurring antibodies through masked language modeling to capture both evolutionary relationships and structural constraints within antibody sequences.[28,32] However, like other current antibody language models, these tools face a critical limitation: their pretrained embeddings naturally cluster by sequence identity and germline gene usage,[29,31] making them more adept at finding similar sequences than functionally similar antibodies with divergent sequences. This highlights a fundamental need for methods that can specifically learn the complex sequence patterns underlying epitope recognition beyond simple sequence similarity.

Recent advances in machine learning, particularly contrastive learning approaches, offer promising solutions to these limitations. Contrastive learning provides a framework for teaching models to recognize when two examples should be considered similar or different, even when observers see no clear patterns in their features. A useful analogy is signature recognition: while one's signature may vary between years and with different pens, contrastive learning enables machine learning models to recognize the fundamental similarities between signatures and distinguish authentic signatures from forgeries. By applying this approach to antibody analysis, we can explicitly train models to recognize structural or functional epitope similarity even when sequence similarity is low. Using carefully curated training data from structural databases and high-throughput epitope mapping experiments, we demonstrate how this approach can enrich antibody language model embeddings with epitope-specificity information while maintaining their broad understanding of antibody sequence space.

In this work, we address three key challenges in antibody epitope prediction. First, we establish reliable sequence-based thresholds for identifying overlapping-epitope antibodies, providing a simple yet powerful tool for repertoire analysis. Second, we develop and validate a model using the well-characterized severe acute respiratory syndrome coronavirus 2 receptor-binding domain (SARS-CoV-2 RBD), where extensive epitope mapping data enable us to demonstrate how targeted training can overcome the germline bias of current language models. Finally, we present a generalized model capable of predicting epitope relationships across diverse protein families (Pfams), which we validate through the successful identification of antibodies targeting overlapping epitopes with the HIV-1 bNAb 8ANC195, a therapeutic candidate that targets a unique epitope on the HIV-1 envelope (Env) protein. These advances provide a comprehensive framework for computational epitope analysis, offering new possibilities for therapeutic antibody discovery and optimization.

RESULTS

Sequence determinants for overlapping-epitope antibodies

To identify sequence features that reliably predict when antibodies target overlapping epitopes, we initially interrogated antibody sequence identity as a potential determinant. To that end, we focused on two key features of antibody recognition: variable (V) gene usage and CDR3 sequence similarity. We analyzed 1,909 non-redundant human antibodies from the Structural Antibody Database (SAbDab),[33,34] generating approximately 1.8 million pairwise comparisons. These pairs were categorized

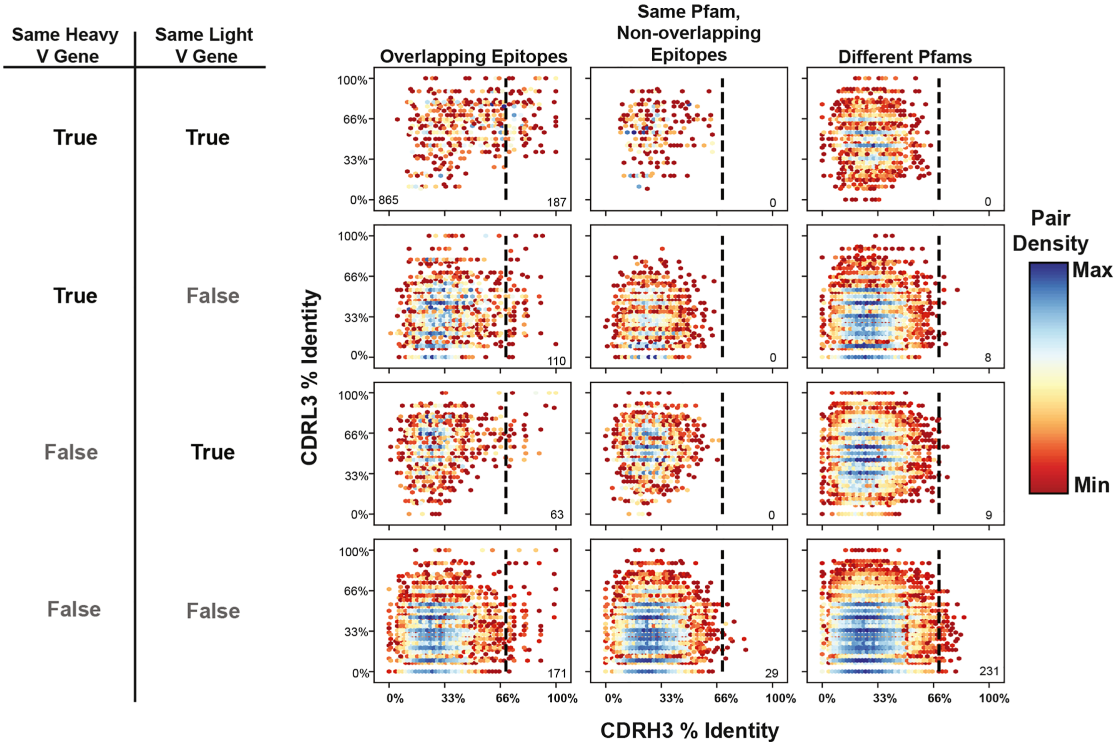

Figure 1. V-gene usage and CDRH3 sequence identity define reliable thresholds for predicting overlapping epitopes

A comprehensive analysis of antibody sequence features predictive of epitope overlap within the Structural Antibody Database (SAbDab). Scatterplots show complementarity-determining region 3 (CDR3) sequence identity relationships between antibody pairs (n = 1,909 antibodies, ~1.8 million pairs) categorized by epitope relationship (columns) and V-gene sharing status (rows). The columns represent overlapping epitopes (left), non-overlapping epitopes on the same protein family (middle), and different protein families (right). Rows indicate V-gene sharing patterns: both heavy and light V genes shared (top), only heavy V gene shared (second), only light V gene shared (third), or neither V gene shared (bottom). The x axis shows CDRH3 amino acid sequence identity, and the y axis shows CDRL3 amino acid sequence identity. Data density is represented by hexagonal binning with color scaling from minimum (dark red) through yellow to maximum density (dark blue). Dashed vertical lines indicate the 70% CDRH3 identity threshold. Numbers in the bottom corners indicate pair counts within the half designated by the line.

based on both their V-gene sharing patterns and binding properties, specifically examining (1) pairs binding overlapping epitopes, (2) pairs binding non-overlapping epitopes within the same Pfam,[35] and (3) pairs binding different Pfams (Figures 1, S1A, and S1B).

Our analysis revealed a hierarchical relationship between sequence features and epitope overlap. For antibody pairs sharing both heavy- and light-chain V genes, we identified a heavy-chain CDR3 (CDRH3) amino acid identity threshold of 70% that serves as a virtually perfect predictor—all pairs exceeding this threshold bound overlapping epitopes within the same Pfam (Figure 1, top row). This predictive power persisted when antibodies shared only one V gene, albeit with an important caveat (Figure 1, rows 2 and 3): while pairs exceeding the CDRH3 threshold and targeting the same Pfam consistently bound overlapping epitopes, 17 of 190 antibody pairs that exceeded this threshold bound antigens from entirely different Pfams. This distinction suggests that when both the heavy- and light-chain germline V genes are shared, this provides additional constraints on antigen specificity beyond epitope recognition patterns.

While these sequence-based rules provide a clear framework for predicting epitope overlap, they also have two major limitations. First, the most predictive rule applies only to the small subset of antibody pairs sharing both V genes. Second, even within this subset, the threshold fails to identify 82% of antibody pairs that do bind overlapping epitopes, resulting in a high false negative rate. These limitations suggest that while sequence identity can provide absolute confidence in some cases, more sophisticated computational approaches may be needed for broader applicability in therapeutic antibody discovery.

To address these limitations, we next explored the ability of antibody large language models to learn the rules of epitope specificity. We focused on two domains: learning discrete epitope bins within one antigen and learning continuous epitope information across diverse Pfams. These approaches, detailed in the following sections, demonstrate how modern computational methods can overcome the constraints of simple sequence-based rules.

Contrastive learning enables epitope-specific encoding of SARS-CoV-2 RBD antibodies

While sequence-based thresholds provide reliable predictions in specific cases, their limited applicability motivated us to develop more sophisticated approaches for predicting epitope relationships. We leveraged the extensive epitope mapping data

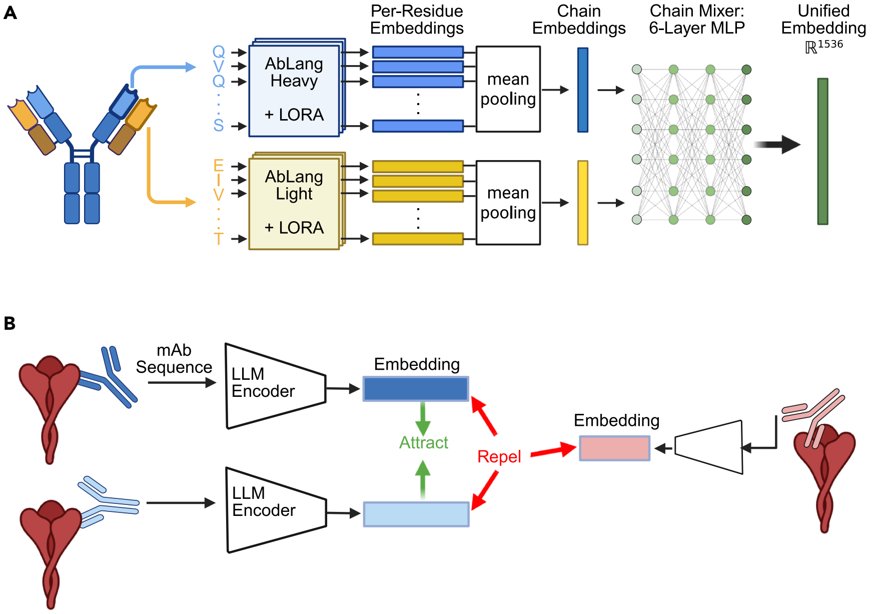

Figure 2. Contrastive learning framework for encoding epitope-specificity information in antibody sequence embeddings

(A) Model architecture for AbLang-RBD and AbLang-PDB and their embedding mechanism. This framework uses frozen AbLang heavy and light models with unfrozen low-rank adaptation parameters, as well as an added 6-layer multi-layer perceptron (MLP) network to create a unified 1,536D embedding for each antibody fed into the model.

(B) Representation of the contrastive learning approach for the "LLM encoder" framework depicted in (A). During training, embeddings of antibodies binding overlapping epitopes (two blue antibodies) are pulled closer together in representation space, while embeddings of antibodies binding non-overlapping epitopes (pink compared to blue) are pushed apart.

available for SARS-CoV-2 RBD antibodies[36–38] to develop and validate a contrastive learning framework that could encode epitope-specificity information directly into antibody sequence embeddings (Figures 1 and 2).

Our model, AbLang-RBD, builds upon the established AbLang-Heavy and AbLang-Light chain language models through targeted fine-tuning using a supervised contrastive learning framework.[28,39–41] The architecture processes paired antibody sequences through a dual-stream transformer network—with 12 separate transformer blocks per chain—followed by a six-layer multi-layer perceptron that generates unified sequence embeddings (Figure 1A). We optimized these embeddings using supervised contrastive learning as described by Khosla et al.,[41] which simultaneously processes multiple positive examples within each training batch, allowing the model to learn from groups of antibodies targeting the same epitope rather than individual pairs (Figure 1B). By training on same-epitope antibodies that fall outside our previously established V-gene and CDRH3 identity thresholds, the model learns new antibody sequence patterns indicative of shared epitope binding that are missed by our outlined V-gene and CDRH3 thresholds.

We trained the model using a previously characterized set of 3,041 SARS-CoV-2 RBD antibodies binned into 12 epitopes based on deep mutational scanning (DMS) results.[37,38] Antibodies were clustered based on heavy V-gene and CDRH3 identity, and clusters were split between training and test datasets using a CDRH3 cutoff of 65%. Despite these strict criteria, visualizing the cosine similarity distributions reveals that our contrastive learning approach effectively distinguishes epitope-bin information (Figure 3A). The pretrained AbLang model showed poor separation between same-epitope and different-epitope pairs (56.0% balanced accuracy), whereas AbLang-RBD improved this distinction, achieving 74.4% balanced accuracy on test set comparisons and 82.7% when comparing test antibodies to the training set.

The effectiveness of our epitope-specific encoding was further demonstrated through dimensionality reduction analysis. t-Distributed stochastic neighbor embedding (t-SNE) visualization[42] reveals that while the pretrained model's embeddings show minimal epitope-based clustering (31.2% *k*-means accuracy),[43] AbLang-RBD achieves near-perfect clustering of training data (99.6%) and substantially improved clustering of test data (54.6%) (Figure 3B). Notably, when test antibodies were misclassified, 43% of errors still placed them within the correct RBD epitope class (out of 4 generally accepted classes),[44] suggesting the model captures meaningful spatial relationships between epitopes despite this information not being provided in training.

To validate that our model learned genuine epitope-specificity information rather than arbitrary clustering, we evaluated its performance against two continuous data sources. First, we examined correlation with the underlying DMS data by obtaining a "center of mass" for each antibody's escape data on the RBD structure (Protein Data Bank, PDB: 8SGU).[45] For each antibody, all residues were assigned a "mass" or "weight" at the location of their α carbon, and then a weighted average was taken over all residues (Figure S1C). AbLang-RBD embeddings exhibited greater correlation with these spatial coordinates (Spearman's $\rho = -0.39$, $p < 5e{-}300$) compared to the pretrained model ($\rho = -0.08$, $p < 5e{-}300$) for the training set as well as for the test set antibodies ($\rho = -0.21$, $p = 5.2e{-}149$; Figure 3C). Second, we assessed performance on an independent set of 237 RBD-specific antibodies with structural epitope information from the PDB (Figures 3D and S1A). The correlation between embedding similarities and buried surface area (BSA) overlap improved dramatically (Spearman ρ: 0.1 to 0.25; p: $8e{-}65$ to $<5e{-}300$). Our binary training paradigm (same versus different epitope bins) generated a bimodal distribution in the embedding space, enabling threshold-based classification: a cosine similarity > 0.85 distinguished highly overlapping epitopes (BSA > 750 Å^2) from non-overlapping pairs with 97% accuracy and specificity (Figure 3D, right). This demonstrates that AbLang-RBD acquired a nuanced understanding of epitope relationships beyond

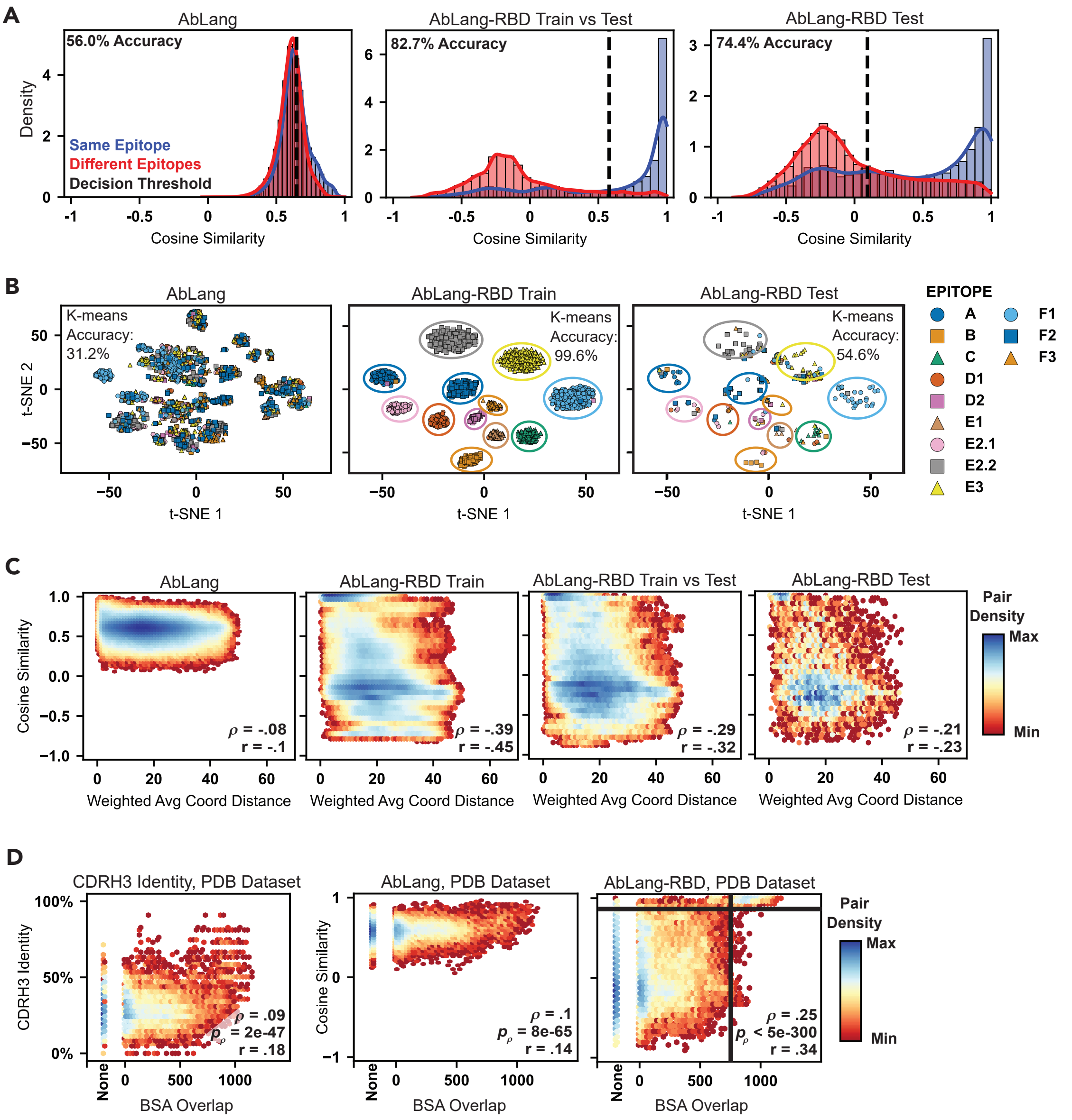

Figure 3. AbLang-RBD learns to predict epitope relationships from binned deep mutational scanning data

(A) Distribution of cosine similarities between antibody pairs binding the same (blue) or different (red) epitopes. The pretrained model's performance is shown on the left, while the fine-tuned model's performance is shown for either the comparisons of train-to-test antibodies (middle) or comparisons of test versus test antibodies (right). Optimal decision thresholds (dashed lines) were determined using validation data.

(B) t-SNE visualization of antibody embeddings colored by epitope class. These are split to show pretrained embeddings (left) or fine-tuned embeddings on train (middle) and test (right) antibodies.

(C) Model performance assessed against continuous deep mutational scanning (DMS) data. Scatterplots show the relationship between antibody pair cosine similarities (y axis) and distance between weighted average spatial coordinates derived from DMS escape maps (x axis). Hexagonal bins are colored by pair density from minimum (dark red) to maximum (dark blue). Spearman's (ρ) and Pearson's (r) correlation coefficients are shown.

(D) Validation using structural data from the Protein Data Bank (PDB). Scatterplots compare CDRH3 sequence identity (left), pretrained AbLang (middle), and AbLang-RBD (right) against buried surface area (BSA) overlap between antibody pairs. Spearman's (ρ) and Pearson's (r) correlation coefficients are shown; p values (p_ρ) shown correspond to the Spearman correlation. Threshold lines are drawn at a cosine similarity of 0.85 and a BSA overlap of 750 Å^2.

discrete bin classifications and also suggests the predicted BSA overlap or relative overlap as an appropriate tunable parameter for our search framework.

These results demonstrate that supervised contrastive learning can effectively encode epitope-specificity information into antibody embeddings, establishing a powerful new framework for computational antibody analysis. While the model shows some limitations in distinguishing relative distances between non-overlapping epitopes, as evidenced by the presence of discrete bands in cosine similarity distributions rather than a continuous gradient (Figure 3C), its ability to accurately identify antibodies targeting shared epitopes represents a significant advance over existing

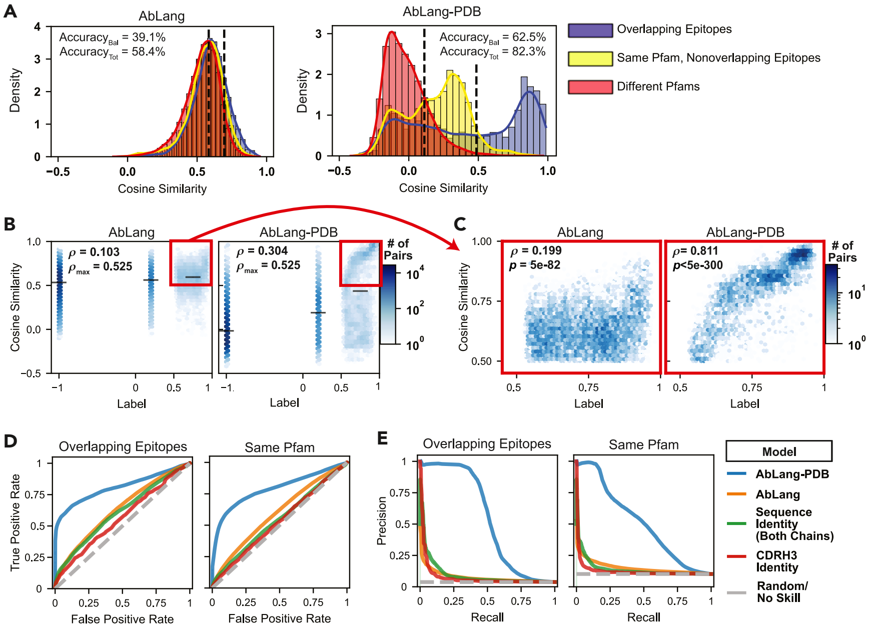

Figure 4. AbLang-PDB enables accurate prediction of epitope relationships across diverse protein families

Evaluation of AbLang-PDB's performance on the Structural Antibody Database (SAbDab), comparing antibodies in the training dataset to held-out antibodies.

(A) Distribution of cosine similarities between antibody pairs categorized as overlapping epitopes (blue), non-overlapping epitopes on the same protein family (yellow), or different protein families (red). Both the pretrained (left) and fine-tuned (right) model results are shown, as well as optimal classification thresholds shown as dashed lines.

(B) Relationship between model-predicted cosine similarities and ground-truth labels. Hexagonal bins colored by pair density (white to dark blue). Black bars indicate mean ± 95% confidence intervals. Spearman correlations (ρ) and maximum possible correlations (ρ_{max}) are shown.

(C) A zoomed-in view of high-confidence predictions for overlapping-epitope pairs (cosine similarity and label $\geq$ 0.5). The Spearman corelation (ρ) and corresponding p value (p) are shown.

(D and E) Receiver operating characteristic curves (D) and precision-recall curves (E) for several classification methods, both for predicting overlapping epitopes (left) and for binding the same protein family (right).

sequence-based methods. This capability, validated against both DMS and structural data, provides a valuable new tool for therapeutic antibody discovery, particularly in cases where traditional sequence similarity metrics fail to identify functionally related antibodies. Most importantly, this framework establishes a foundation for developing even more sophisticated models that can capture the continuous nature of epitope relationships across diverse antigen families.

Developing a generalized model for continuous epitope overlap prediction

To extend beyond predictions for a single antigen, we developed AbLang-PDB, a model capable of predicting the degree of epitope overlap for antibodies targeting antigens from diverse Pfams represented in the PDB.[46–48] Unlike AbLang-RBD's discrete epitope-binning approach, AbLang-PDB employs a regression framework to predict relative degrees of epitope overlap. We maintained the same dual-stream transformer architecture (Figure 1A) but modified the training objective to a mean squared error loss function to optimize for accurate comparisons between unseen antibodies and those in our curated training set.

The training data encompassed 1,517 antibodies spanning 250 Pfams, with relationships between antibody pairs encoded on a continuous scale. Antibodies targeting different Pfams received a label of −1, while those binding non-overlapping epitopes within the same Pfam were assigned 0.2. For antibodies exhibiting epitope overlap, we assigned continuous labels from 0.5 to 1.0 based on their relative degree of structural overlap (Figures S1A and S1B). This labeling strategy was chosen based on the heuristics that antibodies binding the same antigen at non-overlapping sites often have more in common than antibodies that cannot recognize the same antigen; the wide range from 0.5 to 1 for overlapping-epitope antibody pairs was chosen to allow for more separation in the embedding space between those with highly or marginally overlapping epitopes, reflecting our own observations that sequence features converge more when the epitopes exhibit greater overlap.

Antibodies were split between training and test sets as done for AbLang-RBD. Analysis of sequence similarities between training and test sets confirms minimal overlap between them (Figure S2). The impact of this training approach is evident in the distribution of cosine similarities across different antibody pair categories (Figure 4A). While the pretrained AbLang model showed minimal separation between the three categories (39.1% balanced accuracy), AbLang-PDB achieved clear differentiation (62.5% balanced accuracy), with overlapping-epitope pairs predominantly exhibiting cosine similarities above 0.75. More importantly, the model demonstrated a strong correlation with ground-truth labels across all comparisons (Figure 4B). To contextualize this value, we calculated the maximum possible Spearman correlation (ρ_{max} = 0.525) achievable given the large number of ties in our semi-continuous labeling scheme; our model therefore captures a substantial portion of the learnable sequence-epitope relationship.

The model's predictive power is most pronounced for high-confidence predictions among overlapping-epitope pairs. When restricting the analysis to pairs where both the predicted cosine similarity and the ground-truth label are above 0.5, the correlation between the model's prediction and the actual degree of epitope overlap increases dramatically to ρ = 0.811 (Figure 4C, p < 5e−300). This indicates that the model is highly reliable for ranking the extent of epitope overlap among the most promising candidates.

Comprehensive benchmarking revealed substantial improvements over existing methods (Figures 4D–4F; Table 1). For overlapping-epitope classification, AbLang-PDB achieved an area

CellPress
OPEN ACCESS

Table 1. Benchmarked model performance

		SARS-CoV-2 RBD DMS dataset			SAbDab					
		Same epitope			Same Pfam			Overlapping epitopes		
Pairs used	Model	AUROC	Avg Prec	F1	AUROC	Avg Prec	F1	AUROC	Avg Prec	F1
Train versus test	**AbLang-RBD**	**0.84**	**0.64**	**0.59**	0.51	0.15	0.19	0.58	0.16[a]	0.14
	**AbLang-PDB**	0.54	0.15	0.20	**0.79**	**0.51**	**0.50**	**0.81**	**0.54**	**0.56**
	AbLangPre	0.57[a]	0.18	0.21[a]	0.59	0.15	0.21	0.63	0.08	0.13
	AbLang-Heavy	0.56	0.16	0.21	0.62	0.18	0.23	0.65	0.10	0.14
	AbLang2	0.54	0.13	0.20	0.54	0.12	0.19	0.54	0.05	0.08
	IgBERT	0.54	0.14	0.20	0.58	0.15	0.21	0.60	0.07	0.12
	BALM	0.56	0.16	0.20	0.59	0.15	0.22	0.61	0.07	0.12
	AntiBERTy	0.57	0.17	0.21	0.64[a]	0.19[a]	0.24[a]	0.66[a]	0.12	0.17[a]
	ESM-2	0.56	0.18	0.20	0.55	0.13	0.19	0.60	0.08	0.10
	Parapred	0.55	0.15	0.20	0.54	0.12	0.19	0.58	0.06	0.10
	SEQID	0.53	0.14	0.20	0.53	0.12	0.18	0.56	0.07	0.08
	CDRH3ID	0.56	0.19[a]	0.21	0.54	0.14	0.18	0.61	0.09	0.12
Test versus test	AbLang-RBD	**0.73**	**0.39**	**0.39**	0.53	0.21	0.19	0.61	0.25	0.22
	AbLang-PDB	0.53	0.14	0.20	**0.68**	**0.34**	**0.33**	0.68	**0.33**	**0.35**
	AbLangPre	0.57[a]	0.16	0.20	0.63	0.25	0.20	0.68	0.21	0.19
	AbLang-Heavy	0.56	0.15	0.20	0.64	0.23	0.26[a]	0.68	0.19	0.23
	AbLang2	0.53	0.12	0.20	0.53	0.15	0.20	0.56	0.08	0.12
	IgBERT	0.54	0.13	0.20	0.60	0.20	0.23	0.63	0.14	0.18
	BALM	0.55	0.15	0.20	0.60	0.22	0.24	0.63	0.19	0.19
	AntiBERTy	0.57	0.16	0.21[a]	0.66[a]	0.27[a]	0.25	**0.71**	0.26[a]	0.30[a]
	ESM-2	0.56	0.17	0.20	0.56	0.19	0.20	0.60	0.17	0.20
	Parapred	0.55	0.14	0.20	0.59	0.20	0.21	0.63	0.18	0.15
	SEQID	0.55	0.18[a]	0.19	0.58	0.23	0.20	0.69[a]	0.26	0.13
	CDRH3ID	0.53	0.13	0.20	0.54	0.20	0.20	0.64	0.23	0.17

Performance metrics across all models for the SARS-CoV-2 RBD DMS dataset and the SAbDab dataset (subdivided into "same Pfam" and "overlapping epitopes" tasks). Models are evaluated under two conditions: "train versus test" (comparing antibodies in the train dataset to the test dataset) and "test versus test" (comparing held-out antibodies against each other). The bold formatting indicates the best performance in a benchmark. Baseline models include AbLang-Heavy (Olsen et al.[28]); AbLang-Pre, which is a concatenation of AbLang-Heavy and AbLang-Light embeddings (Olsen et al.[28]); AbLang2 (Olsen et al.[29]); IgBERT (Kenlay et al.[30]); BALM (Burbach and Briney[31]); AntiBERTy (Ruffolo et al.[27]); ESM-2 (Lin et al.[25]); and Parapred (Liberis et al.[26]). In these benchmarks, Parapred was used by generating antibody-specific embeddings in a custom method. The final hidden state of the model was extracted and averaged over all CDR residues in both chains to obtain the embedding. AUROC, area under the receiver operating curve; Avg Prec, average precision; RBD, receptor-binding domain, DMS, deep mutational scanning; SEQID, total amino acid sequence identity over the variable region; CDRH3ID, heavy chain complementarity-determining region 3 amino acid sequence identity.
[a]The second best performance in a benchmark.

under the receiver operating characteristic curve (AUROC) of 0.81, an average precision of 0.54, and an F1-score of 0.56, significantly outperforming both the pretrained model (0.63, 0.077, and 0.12) and sequence-identity-based predictions (0.56, 0.08, and 0.08). Similar improvements were observed for Pfam prediction, with 1.23×, 2.69×, and 2.14× enhancements over the best alternative classifier in the respective categories (Figures 4D–4F). The performance profile seen for AbLang-PDB reflects our optimization strategy, where AbLang-PDB was specifically trained to maximize accuracy when comparing novel antibodies against reference sequences in the training set rather than for direct comparisons between entirely unseen antibodies. While this approach led to less substantial performance gains in test-versus-test evaluations (Table 1), the model is still the best in 5/6 categories among these evaluations, with significant improvements over the pretrained model in all of these categories. More importantly, this training paradigm directly supports our intended application of mining large sequence databases for antibodies with overlapping epitopes relative to known therapeutic references.

These results demonstrate that our continuous learning approach successfully captures epitope relationships across diverse Pfams while maintaining high precision for overlapping-epitope predictions. The model's ability to provide reliable confidence scores through cosine similarities makes it particularly valuable for therapeutic antibody discovery, where false positives can be costly.

Experimental validation of AbLang-PDB through epitope-targeted HIV-1 antibody discovery

To validate AbLang-PDB's practical utility, we applied it to identify antibodies sharing epitope overlap with the HIV-1 bNAb

A

Name	8ANC195 Cosine Similarity	8ANC195 % Identity: Heavy Chain	8ANC195 % Identity: Light Chain	Gene Usage: VH	Gene Usage: JH	Gene Usage: VK/L	Gene Usage: JK/L	Germline % Identity: VH	Germline % Identity: VL	CDR Information: CDRH3	CDR Information: CDRL3	CDRH Lengths	CDRL Lengths
**2723-3055 (1-0)**	0.655	37	48	IGHV1-2	IGHJ4	IGKV3-20	IGKJ2	70	72	VRGRSCCDGRRYCNGADCFNWDFEH	QCF......EG	8.8.25	7.3.5
**2723-6245 (1-1)**	0.653	35	46	IGHV1-2	IGHJ4	IGKV3-20	IGKJ2	72	76	VRGSSCCGGRRHCNGADCFNWDFQY	QCL......EA	8.8.25	7.3.5
**3602-2 (1-2)**	0.650	38	51	IGHV1-46	IGHJ4	IGKV3-20	IGKJ3	73	86	AKDAGEPGWTAY..RRGYPIFFFDT	QQYVR..TPLT	8.8.23	7.3.9
**5157-3 (1-3)**	0.621	45	39	IGHV1-8	IGHJ3	IGLV2-14	IGLJ3	74	81	AVATI...............SAFDI	SSYIHTGPPWL	8.8.10	9.3.11
**3514-4 (1-4)**	0.619	38	39	IGHV1-2	IGHJ5	IGLV3-10	IGLJ2	77	85	YIFIHWA...........NGYMKDH	YSTHS.SLYSA	8.8.14	6.3.10
**3602-870 (1-5)**	0.610	37	55	IGHV1-46	IGHJ4	IGKV3-20	IGKJ3	72	83	ARDAGERGLR......GYSVGFFDS	HQYG..TTPYT	8.8.19	7.3.9
**2723-6 (1-6)**	0.603	37	45	IGHV1-2	IGHJ1	IGKV3-20	IGKJ2	68	77	ARGKSCCEGRRFCSPNDCYNWDFEH	QFH......EA	8.8.25	7.3.5
**2723-4886 (1-7)**	0.602	33	50	IGHV1-2	IGHJ1	IGKV3-20	IGKJ2	66	75	AMRDYCRDD.......NCNIWDLRH	QHR......ET	8.8.18	6.3.5
**4513-8 (1-8)**	0.582	45	53	IGHV1-69	IGHJ4	IGKV3D-20	IGKJ4	78	86	ARGGFSN............NWLGAY	HQYGF..APLT	8.8.13	7.3.9
**6420-9 (1-9)**	0.567	49	40	IGHV1-69	IGHJ4	IGLV7-46	IGLJ2	85	94	ARETLGYSG........SYGPAFDF	LLSHS..GVPV	8.8.17	9.3.9
8ANC195 (+)	1.000	100	100	IGHV1-69	IGHJ4	IGKV1-5	IGKJ1	53	86	TTTSTYDKWSG...LHHDGVMAFSS	QQYDT..YPGT	9.7.25	7.3.9
VRC01	0.534	39	58	IGHV1-2	IGHJ2	IGKV3-20	IGKJ2	69	64	TRGKNCD...........YNWDFEH	QQY......EF	8.8.14	6.3.5

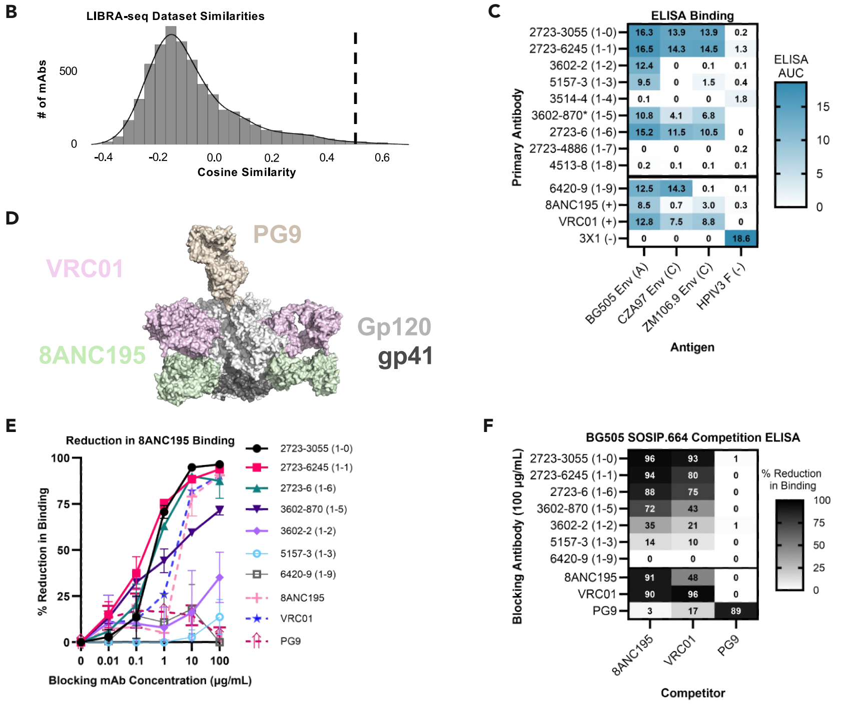

Figure 5. AbLang-PDB successfully identifies HIV antibodies that compete for binding with 8ANC195

Experimental validation of AbLang-PDB predictions using HIV broadly neutralizing antibody 8ANC195.

(A) Sequence characteristics of top candidate antibodies selected by cosine similarity to 8ANC195. The table shows model predictions, sequence identity metrics, gene usage, and CDR information for each antibody. Reference antibodies 8ANC195 and VRC01 are included for comparison.

(B) Distribution of cosine similarities across the complete LIBRA-seq dataset (n = 7,056 antibodies), with dashed line indicating recommended threshold (0.5) for mining overlapping-epitope candidates.

(C) ELISA binding profiles against HIV-1 envelope SOSIP.664 constructs (BG505, CZA97, ZM106.9) and HPIV3 F control protein. Binding strength is indicated by the area under the curve (white to blue). The 3X1 antibody was included as an HPIV3-specific control.

(D) Structural representation of HIV-1 BG505 envelope showing competitor antibody epitopes: 8ANC195 (green, gp120-gp41 interface), VRC01 (pink, CD4-binding site), and PG9 (tan, V1-V2 region) from PDB: 5VJ6, 8VGW. The envelope surface is shown with gp120 (light gray) and gp41 (black).

(E) Competition ELISA curves showing percentage of reduction in binding of biotinylated 8ANC195 (10 μg/mL) to BG505 SOSIP.664 in the presence of increasing concentrations of blocking antibodies. Filled symbols indicate mAbs displaying competition with 8ANC195.

(F) Competition matrix showing percentage of reduction in binding at fixed concentrations (blocking: 100 μg/mL; detection: 10 μg/mL 8ANC195 and 1 μg/mL VRC01 and PG9). Values range from no competition (white) to complete competition (black).

8ANC195.[49–51] This antibody represents an ideal test case due to its unique binding site at the gp120-gp41 interface and its therapeutic potential, despite having somewhat limited neutralization breadth compared to other bNAbs.[52] We analyzed a dataset[53,54] of 7,056 class-switched antibodies from persons living with HIV-1, computing cosine similarities between each antibody and 8ANC195's embedding (Figures 5A and 5B; methods). From this analysis, we identified 20 candidates with the highest cosine similarities (range: 0.567–0.655). After eliminating eight antibodies with high sequence identity to other selected candidates and two showing potential reactivity to respiratory syncytial virus or hepatitis C virus in their linking B cell receptor (BCR) to antigen specificity through sequencing (LIBRA-seq) profiles, we prioritized 10 antibodies for experimental characterization. Notably, the intermediate cosine similarity scores suggested partial rather than complete epitope overlap, consistent with 8ANC195's unique epitope characteristics (Figure 5A).

We first assessed HIV-1 specificity through ELISA against three diverse HIV-1 Env SOSIP.664 constructs, BG505 (clade A), CZA97 (clade C), and ZM106.9 (clade C), using human parainfluenza virus 3 fusion (HPIV3 F) protein as a negative control (Figures 5C, S3A, and S3B[53]). Seven of the ten selected antibodies (70%) demonstrated HIV-1 Env specificity, with six (60%) showing cross-clade binding. Out of our unbiased selection, two had previously been characterized—2723-3055 and 3602-870—both of which had been shown to potently neutralize a broad panel of tier 2 HIV-1 viruses (12/12 and 11/14 viruses tested).[53]

To assess epitope overlap, we performed competition ELISAs against BG505 SOSIP.664 using a panel of well-characterized HIV-1 antibodies targeting distinct epitopes: 8ANC195 (gp120-gp41 interface),[51] VRC01 (CD4 binding site),[55] and PG9 (V1-V2 region)[56] (Figures 5D–5F and S3C). Five antibodies (50%) showed competition with 8ANC195, defined as having >30% reduction in binding, with four of those displaying strong competition (>70% reduction). These same antibodies also competed with VRC01 at a slightly weaker level but not with PG9, consistent with the structural overlap between the 8ANC195 and VRC01 epitopes.

The success rate of our computational predictions—50% for identifying 8ANC195-competing antibodies and 70% for HIV-1

CellPress
OPEN ACCESS

Env specificity—highlights AbLang-PDB's potential to streamline therapeutic antibody discovery by accurately identifying functionally relevant candidates that conventional sequence similarity metrics can miss. This is particularly noteworthy given that the model identified two previously validated bNAbs without any prior knowledge of their functional properties. These results support AbLang-PDB's utility for therapeutic antibody discovery, especially in cases where conventional sequence similarity metrics would fail to identify functionally related candidates.

DISCUSSION

The identification of antibodies targeting overlapping epitopes remains a critical challenge in therapeutic antibody development. Current approaches typically rely on experimental screening[57] or database searches for sequence-similar antibodies,[7] both of which have significant limitations. Our work establishes three complementary computational strategies that address this challenge at different levels of complexity and applicability.

First, we provide rigorously validated sequence-identity-based thresholds for predicting epitope overlap. When antibody pairs share both heavy- and light-chain V genes and have CDRH3 amino acid identity exceeding 70%, they not only consistently bind overlapping epitopes but are also guaranteed to target the same Pfam. This constraint relaxes slightly when only one V gene is shared—while the 70% CDRH3 identity threshold still perfectly predicts overlapping epitopes when antibodies target the same Pfam; some pairs meeting this criterion may bind antigens from different Pfams altogether. While these findings appear straightforward, they represent the first systematic validation of such thresholds across more than 200 antigen specificities. These simple yet powerful criteria provide immunologists with reliable tools for initial repertoire analysis, particularly valuable when analyzing vaccine responses or comparing antibody lineages across individuals.

Second, through our first model, AbLang-RBD, we demonstrate how supervised contrastive learning can enhance language model embeddings with epitope-specificity information. Using the well-characterized SARS-CoV-2 RBD as a model antigen, we showed that this approach achieved 74.4% accuracy in predicting epitope relationships between previously unseen antibodies. The model's ability to generalize beyond its training data is evidenced by its improved correlation with both DMS data and structural epitope measurements compared to sequence-based metrics or the pretrained model. This indicates that our contrastive learning framework indeed captures genuine epitope-specificity information rather than merely clustering similar sequences.

Third, we developed AbLang-PDB, which extends epitope prediction capabilities across diverse Pfams while capturing continuous relationships between epitopes. This model demonstrates substantial improvements over existing methods, achieving significant increases in AUROC, average precision, and F1-scores for both same-antigen Pfam pair predictions and overlapping-epitope prediction across a wide range of benchmark models (Figures 4D and 4E; Table 1). Notably, our contrastive learning approach increased average precision 7-fold relative to the pretrained model and 4.5-fold relative to the second-best-performing model, AntiBERTy (Figure 4E; Table 1). Beyond classification performance, AbLang-PDB accurately estimates epitope overlap through cosine similarity scores, with high-confidence predictions (cosine similarity > 0.5) for overlapping-epitope antibody pairs showing strong correlation ($\rho = 0.811$) with actual epitope overlap (Figure 4C).

Our model demonstrated therapeutic relevance through the identification of HIV-specific antibodies sharing epitope overlap with 8ANC195. Of our 10 computationally selected candidates, 70% showed HIV-1 specificity, 50% competed with 8ANC195 for binding, and 20% were broadly neutralizing. The 2 bNAbs had both been previously discovered, but of note, they were the only two previously discovered bNAbs present among the 7,056 mAbs in the database. Feature attribution analysis using integrated gradients of the five experimentally validated 8ANC195-competing antibodies revealed unexpected insights into AbLang-PDB's decision-making process (Figure S4).[58] The analysis showed that predictions were predominantly driven by light-chain features, particularly residues surrounding the CDRL3 loop. We then repeated this analysis over the broader set of all test antibodies (see methods for full details). The attribution pattern was consistent with 8ANC195-competing antibodies, showing that light-chain residues received significantly higher attribution scores than heavy-chain residues, with the exception that there was considerably greater variability in results between antibodies than seen for the set of 5 antibodies. These findings suggest that while heavy chains are traditionally considered the primary determinants of binding specificity, machine learning approaches may reveal previously underappreciated contributions of light-chain sequences to epitope recognition patterns.

Beyond epitope overlap prediction, our models can be leveraged for large-scale antigen-specificity screening applications as was seen by our high hit rate for HIV-1-specific antibodies. One implementation to capture a wider diversity of epitopes during antigen-specificity screening utilizes a multi-reference search approach. Here, a panel of antibodies with known antigen specificity is used as a reference, followed by classification of antigen specificity based on having cosine similarities of >0.2 compared to the majority of reference antibodies.

The selection between AbLang-RBD and AbLang-PDB depends on two critical considerations: existing knowledge of antigen specificity and extent of epitope overlap. For antibodies with confirmed SARS-CoV-2 RBD binding, AbLang-RBD is recommended. This is due to its specialized training on extensive RBD-specific data, achieving superior performance across all evaluated metrics (AUROC, average precision, and F1-scores) on SARS-CoV-2 datasets compared to AbLang-PDB. However, for antibodies lacking confirmed RBD specificity or when continuous epitope overlap quantification is prioritized, AbLang-PDB provides broader applicability with more correlated cosine similarity values to relative epitope overlap measures, whereas AbLang-RBD exhibits more discrete, bimodal similarity distributions.

However, our approaches have important limitations. The sequence-identity-based thresholds, while providing perfect precision for predicting overlapping epitopes, exhibit low recall—failing to identify 82% of antibody pairs that do share epitope overlap. AbLang-RBD demonstrates high performance on SARS-CoV-2 index strain RBD epitope prediction

but faces two key constraints: it is not clear that it will generalize to RBD-specific antibodies incapable of binding the index strain and its training approach using epitope bins from DMS data has not been validated to show that this will work using epitope bins from the more commonly available antibody-antibody competition binding data. AbLang-PDB's training paradigm is optimized for comparing novel antibodies against reference antibodies from its training set rather than directly comparing two previously unseen antibodies. Additionally, this training paradigm is likely influenced by the specific training labels chosen by hand (−1, 0.2, and 0.5–1.0), which represent heuristic choices that could potentially be optimized through systematic hyperparameter search approaches. Furthermore, while our HIV-1 validation demonstrates practical utility, this antigen is well represented in our training data, though notably, our validation epitope (8ANC195's epitope) had minimal representation.

Despite these limitations, our work provides a comprehensive framework for computational epitope analysis that will be of significance for the field of therapeutic antibody discovery. The combination of simple sequence rules and sophisticated machine learning models offers researchers a tiered approach to identifying overlapping-epitope antibodies, from rapid initial screening to detailed prediction of epitope relationships. Looking forward, these methods can be further enhanced through integration with emerging structural prediction tools and expanded training datasets, potentially enabling even more accurate prediction of antibody-antigen interactions.

METHODS

Data curation

We curated a comprehensive dataset from the SAbDab[33,34] (February 19, 2024, cutoff date) for training and validating the AbLang-PDB model. Starting with 16,105 antibody-antigen complexes, we applied the following filtering criteria: resolution ≤ 4.5 Å, human antibodies with both chains present, and ≥5 amino acid differences between antibodies. This yielded 1,909 non-redundant complexes, of which 184 had no same-Pfam pairs and 485 had no overlapping-epitope pairs.

Antigen classification utilized pfam_scan[35,59] software to group antigens by domain architecture using hidden Markov models. Multiple Pfam assignments were consolidated such that any shared Pfam between antigens classified their respective antibodies as targeting the "same Pfam" and thus a machine learning label of 0.2 or greater. When no overlap was present, these pairs were assigned a machine learning label of −1. For quantifying epitope overlap, we employed two complementary approaches.

Approach 1: BSA

Here, we calculated BSA per residue using the Shrake-Rupley[60] algorithm within the Dictionary of Secondary Structure Predictions (DSSP 4.0)[61,62] tool. This algorithm calculates solvent accessibility in a method equivalent to rolling a ball the size of a water molecule along a protein surface and calculating the square angstroms for each residue that are accessible to this water molecule. To translate the accessible surface area (ASA) into antibody BSA, the amount of surface area at each residue for the antigen is calculated either in complex with the antibody or after synthetically removing the antibody from the structure file, such that $BSA_{residue\_i} = ASA_{residue\_i,Ag\ only} - ASA_{residue\_i,Ag\ in\ complex}$. Per-residue BSA overlap between two antibody-antigen complexes was calculated as

$$BSA\_OVERLAP_{residue\_i} = min\left(BSA_{residue\_i,complex\_1}, BSA_{residue\_i,complex\_2}\right),$$

where *residue_i* refers to equivalent residues in each of the 2 complexes as defined by a pairwise sequence alignment of the antigen sequences. Pairwise sequence alignments of the antigen sequences were accomplished using the BLOSUM62 matrix[63] and Needleman-Wunsch[64] algorithm. Antibody pairs with total BSA overlap summed over all residues ≤20 Å^2 were labeled as non-overlapping (Figure S1A, label = 0.2).

Approach 2: Distance and volume overlap

In this approach, we defined epitopes as antigen heavy atoms within 4.5 Å of antibody atoms, and these selections were accomplished using PyMOL.[65] For all antibody-antigen complexes sharing at least one Pfam label, the antigens were aligned in PyMOL. Next, the volume overlap of epitope atoms was calculated using PyMOL's overlap function, with pairs showing overlap of ≤5 Å^3 labeled as non-overlapping (label = 0.2).

For overlapping epitopes, final labels were assigned on a continuous scale from 0.5 to 1.0 using the formula

$$label = min\left(1, 0.5 + (rBSA\_OVERLAP + rATOM\_OVERLAP)^{0.75}\right),$$

where *rBSA_OVERLAP* and *rATOM_OVERLAP* represent overlap relative to the smaller of the two self-overlap values seen for each epitope pair. For partitioning antibodies between datasets, antibodies sharing both heavy and light V genes and CDRH3 amino acid identity >65% were assigned to the same clone group. These groups were then distributed across training (80%), validation (10%), and test (10%) sets, ensuring no clone group appeared split between multiple sets. Additionally, pairs with >92.5% sequence identity in either chain were excluded to maintain diversity.

For AbLang-RBD, we utilized published DMS data comprising 3,195 antibodies from 2 papers,[37,38] of which only the 3,093 that demonstrated binding to the SARS-CoV-2 index strain were kept. These antibodies were clustered based on heavy-chain V-gene usage and CDRH3 amino acid identity >70%, with clusters distributed across training (80%), validation (10%), and test (10%) sets such that no antibodies in the same cluster existed in the training and test sets. A separate test set was curated from the PDB by selecting RBD-specific antibodies from the Coronavirus Antibody Database (CoV-AbDab)[36] that demonstrated index strain binding and were unique from those in the DMS dataset. This left 237 antibodies and 27,345 pairs.

Model architecture

We built our approach upon the pretrained AbLang framework, which comprises separate heavy- and light-chain transformer models for antibody sequence analysis. We utilized the published AbLang model weights from Huggingface (qilowoq/AbLang_heavy and qilowoq/AbLang_light), accessed through the transformers library (AutoModel and AutoTokenizer).[28,66]

The base architecture follows RoBERTa[67] with modifications for antibody sequence processing: each chain is processed through 12 transformer blocks containing 12 attention heads, with a hidden dimension of 768 and an intermediate dimension of 3,072. A learned positional embedding layer handles sequences up to a length of 160.

For sequence processing, antibody amino acid sequences were first tokenized using the transformers module. Heavy- and light-chain sequences were processed independently through their respective models to generate chain-specific embeddings. For each chain, the final hidden layer outputs (768D vectors) from all non-masked positions were mean pooled. Whereas for the pretrained AbLang model, we simply concatenated these chain embeddings, the architecture for AbLang-RBD and AbLang-PDB introduces additional processing layers to enable cross-chain information flow. Specifically, the concatenated 1,536D vector (768 dimensions per chain) is processed through a 6-layer multi-layer perceptron with rectified linear unit (ReLU) activation between layers, except for the final layer. The normalized output of this network serves as the unified antibody embedding.

To enable efficient fine-tuning while preserving pretrained weights, we employed QLORA (quantized low-rank adaptation) with rank R = 16, alpha = 32, and dropout = 0.3.[39] This dual-stream architecture—with 12 transformer blocks per chain, followed by the cross-chain mixing network—allows the model to capture both chain-specific features and relationships between heavy- and light-chain sequences.

AbLang-RBD training

The AbLang-RBD model was trained using a supervised contrastive learning approach to differentiate antibody embeddings based on their epitope label. Specifically, we employed the supervised contrastive loss function, as introduced by Khosla et al.[41] This loss function is designed to pull embeddings with the same label closer together in the embedding space while pushing apart those with different labels.

Training was performed with a batch size of 256 antibodies. Optimization was performed using the AdamW optimizer with a learning rate of 1e−5. During training, we froze all pretrained weights except for the QLORA adaptation parameters and the six "mixing" layers that enable crosstalk between heavy- and light-chain embeddings. The detailed mathematical formulation of the loss function is outlined below.

Contrastive loss mathematical formulation

Notation:

(1) Batch size of antibodies: $B = 256$.
(2) Embeddings: $z_i \in R^{1,536}$ *for* $i = 1, \ldots, B$.
(3) Epitope labels: $y_i \in Z$ *for* $i = 1, \ldots, B$.
(4) Temperature parameter: $\tau = 0.5$.
(5) Set of positive pairs for anchor antibody i: $\mathcal{P}(i) = \{j | y_i = y_j, j \neq i\}$.

Step 1: Similarity matrix. Compute the similarity matrix $S \in R^{B \times B}$, where each element represents the scaled cosine similarity between two antibody embeddings:

$$S_{ij} = \frac{z_i^T z_j}{\tau}.$$

Step 2: Numerical stability. For numerical stability during exponentiation, subtract the maximum value from each row of the similarity matrix:

$$\overline{S}_{ij} = S_{ij} - \max_k S_{ik}.$$

Step 3: Denominator calculation. For each antibody i, calculate the denominator D_i by summing the exponentiated similarities over all other antibodies in the batch:

$$D_i = \sum_{k=1, k \neq i}^{B} \exp(\overline{S}_{ik}).$$

Step 4: Final loss calculation. The total loss is the average of the negative log likelihood over all positive (same epitope label) pairs in the batch.

The loss contribution from a single positive pair (i,j) is $-\log \frac{\exp(\overline{S}_{ij})}{D_i}$.

The final loss averages these terms:

$$\mathcal{L}_{contrastive} = \frac{1}{\sum_{i=1}^{B} |\mathcal{P}(i)|} \sum_{i=1}^{B} \sum_{j \in \mathcal{P}(i)} \left(-\log \frac{\exp(\overline{S}_{ij})}{D_i} \right).$$

Training proceeded for 400 epochs on a single NVIDIA A6000 GPU, requiring approximately 5 h, including inter-epoch evaluations. Model selection was based on AUROC performance on the validation set (weighted by epitope class size), with the epoch 280 checkpoint achieving optimal performance.

Histogram generation and pairwise accuracy or F1 calculation

Distributions of antibody pair relationships were visualized and analyzed using histograms implemented in Python 3.8.18 with seaborn 0.13.1. All histograms were generated using probability density normalization with 30 uniform-width bins. Classification thresholds were determined differently for AbLang-RBD and AbLang-PDB versus the pretrained model. For AbLang-RBD and AbLang-PDB, thresholds were optimized to maximize balanced accuracy on the validation dataset. The pretrained model threshold in Figure 4A was similarly optimized using train versus validation parameterization, while in Figure 3A, it was optimized for maximal balanced accuracy across the complete dataset (note: this approach overestimates model performance).

For three-category classification, optimal decision boundaries were determined via grid search across 90,000 threshold combinations (300 × 300 cosine similarity values). The threshold pair yielding maximum balanced accuracy across all three categories (overlapping epitopes, non-overlapping epitopes within the same Pfam, and different Pfams) was selected. Balanced accuracy was calculated as the mean of individual category accuracies, in contrast to total accuracy, which can be biased by class imbalance.

Calculation of a representative DMS coordinate

DMS escape data were obtained for 1,375 SARS-CoV-2 RBD antibodies from the publicly available Bloom laboratory database.[37,38,68,69] To translate high-dimensional DMS data into a single, representative 3D coordinate for each antibody's

epitope, we calculated a weighted average of the SARS-CoV-2 RBD's atomic coordinates. The goal was to find the center of mass for each epitope, where the "mass" of each residue is determined by its importance for antibody binding.

For each antibody, we first obtained its complete DMS escape map, which details how every possible mutation to the RBD affects antibody binding. For each residue position on the RBD, we summed the escape scores of all possible mutations at that site. This sum, w_i, serves as a weight representing the overall importance of residue i to the antibody's epitope. We then used the 3D coordinates (x_i) of the α carbon of each residue from the SARS-CoV-2 RBD structure (PDB: 8SGU). The final 3D epitope coordinate (x) was calculated as the weighted average of these α carbon positions:

$$\bar{x} = \frac{\sum_i w_i x_i}{\sum_i w_i}.$$

This method provides a single 3D point for each antibody's epitope, allowing for the calculation of simple Euclidean distances between epitopes for comparison with our model's predictions (Figure S1C).

Regression analysis

Statistical analyses were performed using SciPy (v.1.10.1) for correlation calculations and significance testing.[70] Spearman's rank correlation (spearmanr) and Pearson correlation (pearsonr) coefficients were calculated for various pairwise comparisons. In Figure S1B, the relationship between relative BSA (rBSA) and training labels was fit using linear regression (scipy.stats.linregress), excluding pairs with labels below 0.5. For correlation analyses in Figures 4D, 5B, and 5C, Spearman correlations were calculated with associated p values; p values below the numerical precision limit of 64-bit floating point numbers are reported as $p < 5e{-}300$.

For Figure 4B, we calculated the maximum achievable Spearman correlation (ρ_{max}) by considering the optimal ranking scenario where (1) all antibody pairs with a label of -1 rank below those with a label of 0.2, (2) all pairs with label 0.2 rank below those with labels of $\geq$0.5, and (3) pairs with labels between 0.5 and 1.0 are perfectly rank ordered. Mean values with 95% confidence intervals were calculated for discrete label categories (-1 and 0.2) and for the continuous range of labels $\geq$0.5 (plotted at $x = 0.75$). For Figure 4C, analysis was restricted to pairs with both predicted cosine similarities and ground-truth labels between 0.5 and 1.0 to assess performance on high-confidence predictions.

t-SNE analysis and *k*-means accuracy calculation

Dimensionality reduction and clustering analyses were performed using scikit-learn (v.1.3.2). For t-SNE visualization, 1,536D antibody embeddings were reduced to two dimensions using the following parameters: principal-component analysis (PCA) initialization, automatic learning rate determination, a perplexity of 30, and a maximum of 1,000 iterations with a learning rate of 1,000. For AbLang analysis, the complete dataset was visualized in aggregate. For AbLang-RBD, while dimensionality reduction was performed on the complete dataset, training and test sets were subsequently visualized separately to assess generalization performance.

Clustering analysis was performed using *k*-means with cosine similarity as the distance metric. The algorithm was initialized with 12 clusters using the *k*-means++ strategy for greedy centroid initialization and allowed to run for a maximum of 300 iterations. Clustering accuracy was assessed by assigning the most highly represented epitope class within each cluster as the cluster's representative epitope. Antibodies within each cluster were considered accurately clustered if they matched this epitope and incorrectly clustered otherwise. This approach, while disadvantaging underrepresented epitopes due to class imbalance, provides a conservative estimate of clustering performance.

For visualization clarity, we cycled through three marker shapes (circles, squares, and triangles), as well as ten distinct colors.

AbLang-PDB training

The AbLang-PDB model was trained using the architecture described in the model architecture section, utilizing the curated structural antibody dataset. During training, we maintained the pretrained weights of the base model, modifying only the QLORA adaptation parameters and the six mixing layers responsible for cross-chain information integration. Training employed the AdamW optimizer with a learning rate of $1e{-}5$ and a mean squared error loss function, using a batch size of 16.

To address class imbalance in the training data, we implemented a balanced sampling strategy where each epoch processed 15,270 antibody pairs, evenly distributed across three categories: overlapping epitopes, non-overlapping epitopes within the same Pfam, and pairs targeting different Pfams. While this approach ensured equal representation of each category during training, it resulted in more unique pairs from the non-overlapping-epitope classes being trained on.

Training proceeded for 500 epochs on an NVIDIA A6000 GPU, requiring approximately 36 h, including inter-epoch evaluations. Model selection was based on AUROC performance comparing training and test sets, with the epoch 240 checkpoint achieving optimal performance.

Feature attribution analysis

To interpret which sequence features AbLang-PDB uses to make predictions, we employed the integrated gradients method using the Captum library for PyTorch.[58] We calculated per-residue attribution scores, which quantify how much each amino acid contributed to the final cosine similarity prediction. A pad-token embedding was used as the baseline for all calculations. Two primary analyses were conducted: (1) we calculated attributions for the five experimentally validated 8ANC195-competing antibodies against 8ANC195 itself and (2) we computed attributions for every antibody in the test set against its nearest neighbor (by cosine similarity) in the training set. For visualization, mean attribution scores were mapped onto the 8ANC195 structure (PDB: 7KDE) and plotted by international immunogenetics information system (IMGT) position for both the heavy and light chains.

Receiver operating characteristic, precision-recall, and F1-score calculation

Model performance was evaluated using multiple complementary metrics implemented through scikit-learn. For receiver operating characteristic (ROC) analysis, we calculated true positive and false positive rates across 2,001 equally spaced thresholds spanning the range of possible prediction values (cosine similarity from −1 to 1 for model predictions and from 0 to 1 for sequence identity comparisons). The area under the ROC curve was computed using scikit-learn's trapezoidal rule implementation. For AbLang-RBD, AUROC values were calculated separately for each of the 12 epitope classes and combined using a weighted average based on class size. For AbLang-PDB, the calculation used a binary classification scheme where overlapping-epitope pairs constituted the positive class and non-overlapping pairs the negative class.

Precision-recall characteristics were assessed using scikit-learn's precision_recall_curve and average_precision_score functions. For F1-score calculations, we utilized the previously determined optimal threshold that maximized balanced accuracy. In the case of Pfam classification, F1-scores were calculated considering all antibody pairs targeting the same Pfam as positives, regardless of their specific epitope overlap status.

LIBRA-seq dataset curation

Antibody sequence datasets for 8ANC195-like antibody mining came from previous in-house LIBRA-seq experiments.[53] LIBRA-seq is a high-throughput technology that enables simultaneous identification of antigen specificity and BCR sequences at single-cell resolution. In this approach, B cells are exposed to oligonucleotide-barcoded antigens, allowing quantitative assessment of antigen binding through unique molecular identifiers during subsequent single-cell sequencing. From these experiments, 7,056 class-switched antibody sequences were compiled using peripheral blood mononuclear cells (PBMCs) from persons living with HIV-1.

The dataset comprised 21 LIBRA-seq experiments where antigen-specific B cells were isolated from PBMCs using fluorescence-activated cell sorting (FACS). While this experimental design enriched for HIV-1-specific antibodies in the dataset, the majority of sequences are not expected to be HIV-1 specific. Analysis included only functional, single-cell records from 10× Genomics VDJ sequencing where cells had undergone FACS using PE-labeled antigens, including at least one HIV Env protein and one unrelated control antigen. Each antibody was assigned a unique identifier containing a 4-digit sequencing run prefix, with most run prefixes corresponding to unique donors except for runs 2723 and 3514, which both originated from donor 45 (source of VRC01).[53,71] Nucleotide sequences were processed through IMGT HighV-Quest to determine amino acid sequences, germline gene assignments, CDR3 sequences, and percent identity to germline.[72–75] The resulting amino acid sequences were embedded using the AbLang-PDB model, and cosine similarities were calculated between each antibody and 8ANC195. Selection of the top 20 candidates was performed blind to all functional annotations and specificity data, including suspected antigen specificity toward positive control and negative control antigens, enabling unbiased identification of antibodies with potential epitope overlap based solely on sequence features learned by the AbLang-PDB model.

Antibody production

Antibody heavy and light chains were synthesized as cDNA by Twist Bioscience or Genscript. Variable genes were inserted into either bicistronic plasmids encoding the constant regions of the H chain and either the kappa or lambda light chain or into separate heavy- and light-chain plasmids. mAbs made in house were transiently expressed using the ExpiFectamine transfection reagent (Thermo Fisher Scientific) in Expi293F cells in FreeStyle F17 media supplemented with 0.1% poloxamer 188 and 20% 4 mM L-glutamine (Thermo Fisher Scientific). Transfected cultures were incubated shaking for 5 days at 37°C with 8% CO_2 saturation. After 5 days, cultures were harvested and centrifuged at a minimum of 4,000 rpm for 20 min. Supernatant was then filtered with Nalgene Rapid-Flow disposable filter units with a polyethersulfone (PES) membrane (0.45 or 0.22 μm). Filtrate was run over phosphate-buffered saline (PBS) equilibrated columns containing protein A resin. Columns were then washed with PBS, and purified antibodies were eluted using 10 mL of 100 mM glycine HCL at pH 2.7 into 1 mL of 1 M Tris-HCl (pH 8). These were then buffer exchanged into PBS. The remaining mAbs were synthesized by Genscript in their 10 mL TurboCHO high-throughput antibody expression system.

Antigen production

HPIV3 prefusion stabilized F ectodomain (PDB: 6MJZ)[76] was expressed in Expi293F cells through transient transfection using ExpiFectamine transfection reagents (Thermo Fisher Scientific) in Freestyle F17 expression media (Thermo Fisher Scientific) with the addition of 0.1% pluronic acid F-68 and 20% 4 mM L-glutamine.

Upon transfection, cultures were grown at 37°C and 8% CO_2 saturation levels. Six days after transfection, cultures were centrifuged at 4,000 × g for 20 min and filtered with Nalgene Rapid-Flow disposable filter units with a PES membrane (0.45 or 0.22 μM). Protein was purified through nickel affinity chromatography using an equilibrated, 1 mL, prepacked HisTrap HP column (GE Healthcare, Chicago, IL). The column was equilibrated with 15 mL binding buffer (20 mM sodium phosphate, 0.5M NaCl, and 0.3 M imidazole [pH 7.4]). Purified protein was eluted from the column with 15 mL binding buffer supplemented with 0.5 M imidazole. Concentrated protein was buffer exchanged into PBS. The HisTrap purified protein was further purified by size exclusion on Superose 6 Increase 10/300 GL on the AKTA fast protein liquid chromatography (FPLC) system. Fractions containing pure trimeric HPIV3 were identified through SDS-PAGE and the molecular mass. Antigenicity was confirmed with binding to 3X1. Protein concentration was quantified using UV-visible spectroscopy and frozen at −80°C until use.

HIV-1 Env proteins (BG505, CZA97, and ZM106.9) were designed using the SOSIP platform to yield soluble Env proteins stabilized in the prefusion conformation. These SOSIP constructs incorporated several stabilizing mutations: an

intermolecular disulfide bond between gp120 and gp41 (A501C and T605C), a trimer-stabilizing mutation (I559P), a truncated gp41 transmembrane region at position 664, and an I201C/A433C mutation to inhibit CD4-induced movement of Env. Additionally, a flexible serine-glycine linker was inserted between gp120 and gp41 (positions 507 and 512) to create single-chain constructs.[77]

HIV-1 Env proteins were expressed in a highly similar fashion but with the following caveats. Post-culture and centrifugation, the filtered supernatant was applied to an affinity column of agarose-bound *Galanthus nivalis* lectin (Vector Laboratories) at 4°C. After washing with PBS, proteins were eluted with 30 mL of 1 M methyl-α-D-mannopyranoside. The eluate was buffer exchanged three times into PBS and concentrated using either 30 or 100 kDa Amicon Ultra centrifugal filter units.

Final purification was achieved by size-exclusion chromatography using either a Superose 6 Increase 10/300 GL or Superdex 200 Increase 10/300 GL column on an AKTA FPLC system. Fractions corresponding to correctly folded trimeric Env proteins were collected and validated by SDS-PAGE for molecular weight determination and by ELISA for antigenicity using Env-specific mAbs.

Indirect ELISA

In a 96-well plate, 100 μL of antigen was coated at 2 μg/mL overnight at 4°C. The plates were then washed three times with PBS supplemented with 0.05% Tween 20 (PBS-T) and blocked using 5% bovine serum albumin in PBS. Plates were incubated for 1 h at room temperature and then washed three times using PBS-T. Primary antibodies were diluted in 1% bovine serum albumin in PBS-T starting at 10 μg/mL with a 1:5 dilution. After incubating at room temperature for 1 h and washing with PBS-T, 100 μL of goat anti-human immunoglobulin G (IgG) conjugated to peroxidase was added at a 1:10,000 dilution in 1% bovine serum albumin in PBS-T. These were incubated for 1 h at room temperature, washed three times with PBS-T, and then developed using 3,3′,5,5′ tetramethylbenzidine dihydrochloride (TMB) substrate. Plates were developed for 10 min at room temperature and were then stopped using 1 N sulfuric acid. Absorbance was then measured at 450 nm.

Competition ELISA

Wells of a 96-well plate were coated with 100 μL of 2 μg/mL purified BG505 N332T SOSIP and left at 4°C overnight. Plates were then washed three times using PBS-T, and each well was blocked using 100 μL of 5% bovine serum albumin in PBS for 1 h. After washing three times using PBS-T, primary antibodies were diluted 10-fold starting at 100 μg/mL using 1% bovine serum albumin in PBS-T, and 75 μL was added to each well. After incubating for 1 h at room temperature, without washing, 25 μL of biotinylated antibody was added to each well to final concentrations of 1 and 0.1 μg/mL. This was incubated at room temperature for 1 h and washed three times using PBS-T. Then, 100 μL of streptavidin-horseradish peroxidase (HRP) at a dilution of 1:10,000 in 1% bovine serum albumin in PBS-T was added to each well and incubated for 1 h at room temperature. These plates were then washed three times, and bound antibodies were detected using TMB substrate and sulfuric acid. Competition ELISAs were repeated at least 2 times. Data are displayed as the percentage change in binding relative to the binding of an antibody when no competitor is present.

Structural representation of HIV reference antibodies

A composite image of VRC01, 8ANC195, and PG9 binding BG505 Env was generated by first loading PDB: 5VJ6 into open-source PyMOL Schrodinger, v.2.4.0.[56,65] The antibody-antigen complex was represented as a surface, and PG9 was colored wheat, 8ANC195 light green, gp120 light gray, and gp41 dark gray. PDB: 8VGW was then loaded into PyMOL, and the gp120 structures from one protomer were aligned to that of one gp120 protomer in 5VJ6. VRC01 was then colored pink and shown as a surface without visualization of the Env protein present in its native complex. Finally, ray tracing was performed with default parameters.

Benchmarking baseline models

AUROC, average precision, and F1-scores were calculated as described previously. For the following baseline models, the final residue-level hidden state was averaged over just the heavy chain to generate embeddings: AbLang-Heavy[28] and AntiBERTy.[27] For the following models, the hidden state was averaged over both the heavy and light chains: AbLang2,[29] IgBERT,[30] BALM,[31] ESM-2,[25] and Parapred.[26] For Parapred, this averaging was restricted to CDR residues, with each CDR fed into the model separately, as is standard for this model.[26] For ESM-2, the 650 M parameter model was used, and the heavy and light chains were fed simultaneously, separated by two classification (CLS) tokens as done in Burbach and Briney.[31] For AbLang-Pre, we separately averaged the residue-level embeddings from AbLang-Heavy and AbLang-Light[28] and concatenated them sequentially.

RESOURCE AVAILABILITY

Lead contact

Further information and requests for resources and reagents should be directed to and will be fulfilled by the lead contact, Ivelin S. Georgiev (ivelin.georgiev@vanderbilt.edu).

Materials availability

Materials will be made available upon request under a completed materials transfer agreement (MTA).

Data and code availability

- Sequences for antibodies identified and characterized in this study have been deposited to GenBank with names and corresponding accession IDs as such: 2723-3055 (GenBank: MN580560 and MN580589), 2723-6245 (GenBank: MN580555 and MN580584) 3602-2 (GenBank: PX907376 and PX907377), 5157-3 (GenBank: PX907378 and PX907379), 3514-4 (GenBank: PX907380 and PX907381), 3602-870 (GenBank: MN580654 and MN580665), 2723-6 (GenBank: PX907382 and PX907383), 2723-4886 (GenBank: MN580564 and MN580593), 4513-8 (GenBank: PX907384 and PX907385), and 6420-9 (GenBank: PX907386 and PX907387).
- Associated code for AbLang-RBD and AbLang-PDB is available at https://github.com/IGlab-VUMC/AbLangRBD1 and https://github.com/IGlab-VUMC/AbLangPDB1. Model weights are available at https://huggingface.co/clint-holt/AbLangRBD1 and https://huggingface.co/clint-holt/AbLangPDB1%20 or at Figshare: https://doi.org/10.6084/m9.figshare.29647952.[78]
- Any additional data or code reported in this paper will be shared by the lead contact upon request.

CellPress
OPEN ACCESS

ACKNOWLEDGMENTS

We thank Perry Wasdin for his help in curating antigen-specific training sets that helped to interrogate the efficacy of different models and loss functions and Alexandra Abu-Shmais for her insights on drawing conclusions from pairwise antibody comparisons. We additionally thank Andrea Shiakolas, Ian Setliff, Kelsey Pilewski, Rohit Venkat, and Lauren Walker for LIBRA-seq data. This research was funded, in part, by the Advanced Research Projects Agency for Health (ARPA-H 1AY2AX000077), NIH R01AI175245, and the G. Harold and Leila Y. Mathers Charitable Foundation (MF-2107-01851). The funders had no role in the conceptualization or execution of any studies or drafting of the manuscript. The views and conclusions contained in this document are those of the authors and should not be interpreted as representing the official policies, either expressed or implied, of the US government.

AUTHOR CONTRIBUTIONS

Conceptualization, C.M.H. and I.S.G.; data curation, C.M.H.; formal analysis, C.M.H. and T.M.M.; software, C.M.H. and T.M.M.; methodology, C.M.H. and A.K.J.; investigation, C.M.H., I.S.G., A.K.J., P.A., T.M.M., and P.J.J.; visualization, C.M.H., I.S.G., and A.K.J.; writing – original draft, C.M.H.; writing – review & editing, C.M.H., I.S.G., A.K.J., P.A., T.M.M., and P.J.J.; funding acquisition, I.S.G.; project administration, I.S.G.; supervision, I.S.G.; validation, A.K.J. and P.A.; resources, P.J.J.

DECLARATION OF INTERESTS

I.S.G. is listed as an inventor on patents filed describing antibodies characterized here. I.S.G. is listed as an inventor on the patent applications for the LIBRA-seq technology. I.S.G. is a co-founder of AbSeek Bio. I.S.G. has served as a consultant for Sanofi. The Georgiev laboratory at VUMC has received unrelated funding from Merck and Takeda Pharmaceuticals.

SUPPLEMENTAL INFORMATION

Supplemental information can be found online at https://doi.org/10.1016/j.patter.2025.101419.

Received: March 19, 2025
Revised: September 16, 2025
Accepted: October 14, 2025
Published: November 13, 2025

REFERENCES

1. Lu, R.-M., Hwang, Y.-C., Liu, I.-J., Lee, C.-C., Tsai, H.-Z., Li, H.-J., and Wu, H.-C. (2020). Development of therapeutic antibodies for the treatment of diseases. J. Biomed. Sci. *27*, 1. https://doi.org/10.1186/s12929-019-0592-z.
2. Chames, P., Van Regenmortel, M., Weiss, E., and Baty, D. (2009). Therapeutic antibodies: successes, limitations and hopes for the future. Br. J. Pharmacol. *157*, 220–233. https://doi.org/10.1111/j.1476-5381.2009.00190.x.
3. Mahomed, S. (2024). Broadly neutralizing antibodies for HIV prevention: a comprehensive review and future perspectives. Clin. Microbiol. Rev. *37*, e00152-22. https://doi.org/10.1128/cmr.00152-22.
4. Labrijn, A.F., Janmaat, M.L., Reichert, J.M., and Parren, P.W.H.I. (2019). Bispecific antibodies: a mechanistic review of the pipeline. Nat. Rev. Drug Discov. *18*, 585–608. https://doi.org/10.1038/s41573-019-0028-1.
5. Saphire, E.O., Schendel, S.L., Fusco, M.L., Gangavarapu, K., Gunn, B.M., Wec, A.Z., Halfmann, P.J., Brannan, J.M., Herbert, A.S., Qiu, X., et al. (2018). Systematic analysis of monoclonal antibodies against Ebola virus GP defines features that contribute to protection. Cell *174*, 938–952.e13. https://doi.org/10.1016/j.cell.2018.07.033.
6. Zost, S.J., Gilchuk, P., Case, J.B., Binshtein, E., Chen, R.E., Nkolola, J.P., Schäfer, A., Reidy, J.X., Trivette, A., Nargi, R.S., et al. (2020). Potently neutralizing and protective human antibodies against SARS-CoV-2. Nature *584*, 443–449. https://doi.org/10.1038/s41586-020-2548-6.
7. Olsen, T.H., Abanades, B., Moal, I.H., and Deane, C.M. (2023). KA-Search, a method for rapid and exhaustive sequence identity search of known antibodies. Sci. Rep. *13*, 11612. https://doi.org/10.1038/s41598-023-38108-7.
8. Chen, E.C., Gilchuk, P., Zost, S.J., Suryadevara, N., Winkler, E.S., Cabel, C.R., Binshtein, E., Chen, R.E., Sutton, R.E., Rodriguez, J., et al. (2021). Convergent antibody responses to the SARS-CoV-2 spike protein in convalescent and vaccinated individuals. Cell Rep. *36*, 109604. https://doi.org/10.1016/j.celrep.2021.109604.
9. Chen, E.C., Gilchuk, P., Zost, S.J., Ilinykh, P.A., Binshtein, E., Huang, K., Myers, L., Bonissone, S., Day, S., Kona, C.R., et al. (2023). Systematic analysis of human antibody response to ebolavirus glycoprotein shows high prevalence of neutralizing public clonotypes. Cell Rep. *42*, 112370. https://doi.org/10.1016/j.celrep.2023.112370.
10. Abu-Shmais, A.A., Vukovich, M.J., Wasdin, P.T., Suresh, Y.P., Marinov, T.M., Rush, S.A., Gillespie, R.A., Sankhala, R.S., Choe, M., Joyce, M.G., et al. (2024). Antibody sequence determinants of viral antigen specificity. mBio *15*, e01560-24. https://doi.org/10.1128/mbio.01560-24.
11. Chinery, L., Wahome, N., Moal, I., and Deane, C.M. (2023). Paragraph—antibody paratope prediction using graph neural networks with minimal feature vectors. Bioinformatics *39*, btac732. https://doi.org/10.1093/bioinformatics/btac732.
12. Olsen, T.H., Boyles, F., and Deane, C.M. (2022). Observed Antibody Space: A diverse database of cleaned, annotated, and translated unpaired and paired antibody sequences. Protein Sci. *31*, 141–146. https://doi.org/10.1002/pro.4205.
13. Richardson, E., Galson, J.D., Kellam, P., Kelly, D.F., Smith, S.E., Palser, A., Watson, S., and Deane, C.M. (2021). A computational method for immune repertoire mining that identifies novel binders from different clonotypes, demonstrated by identifying anti-pertussis toxoid antibodies. mAbs *13*, 1869406. https://doi.org/10.1080/19420862.2020.1869406.
14. Wong, W.K., Robinson, S.A., Bujotzek, A., Georges, G., Lewis, A.P., Shi, J., Snowden, J., Taddese, B., and Deane, C.M. (2021). Ab-Ligity: identifying sequence-dissimilar antibodies that bind to the same epitope. mAbs *13*, 1873478. https://doi.org/10.1080/19420862.2021.1873478.
15. Robinson, S.A., Raybould, M.I.J., Schneider, C., Wong, W.K., Marks, C., and Deane, C.M. (2021). Epitope profiling using computational structural modelling demonstrated on coronavirus-binding antibodies. PLoS Comput. Biol. *17*, e1009675. https://doi.org/10.1371/journal.pcbi.1009675.
16. Spoendlin, F.C., Abanades, B., Raybould, M.I.J., Wong, W.K., Georges, G., and Deane, C.M. (2023). Improved computational epitope profiling using structural models identifies a broader diversity of antibodies that bind to the same epitope. Front. Mol. Biosci. *10*, 1237621.
17. Wang, Y., Yuan, M., Lv, H., Peng, J., Wilson, I.A., and Wu, N.C. (2022). A large-scale systematic survey reveals recurring molecular features of public antibody responses to SARS-CoV-2. Immunity *55*, 1105–1117.e4. https://doi.org/10.1016/j.immuni.2022.03.019.
18. Strasser, J., de Jong, R.N., Beurskens, F.J., Wang, G., Heck, A.J.R., Schuurman, J., Parren, P.W.H.I., Hinterdorfer, P., and Preiner, J. (2019). Unraveling the Macromolecular Pathways of IgG Oligomerization and Complement Activation on Antigenic Surfaces. Nano Lett. *19*, 4787–4796. https://doi.org/10.1021/acs.nanolett.9b02220.
19. Goldberg, B.S., and Ackerman, M.E. (2020). Antibody-mediated complement activation in pathology and protection. Immunol. Cell Biol. *98*, 305–317. https://doi.org/10.1111/imcb.12324.
20. Huang, J., Kang, B.H., Ishida, E., Zhou, T., Griesman, T., Sheng, Z., Wu, F., Doria-Rose, N.A., Zhang, B., McKee, K., et al. (2016). Identification of a CD4-Binding-Site Antibody to HIV that Evolved Near-Pan Neutralization Breadth. Immunity *45*, 1108–1121. https://doi.org/10.1016/j.immuni.2016.10.027.
21. Makowski, E.K., Wu, L., Gupta, P., and Tessier, P.M. (2021). Discovery-stage identification of drug-like antibodies using emerging experimental

and computational methods. mAbs *13*, 1895540. https://doi.org/10.1080/19420862.2021.1895540.

22. Liu, C., Denzler, L., Chen, Y., Paige, B., and Martin, A. (2024). AsEP: Benchmarking Deep Learning Methods for Antibody-specific Epitope Prediction. https://doi.org/10.5281/ZENODO.11495514.
23. Eguchi, R.R., Choe, C.A., and Huang, P.-S. (2022). Ig-VAE: Generative modeling of protein structure by direct 3D coordinate generation. PLoS Comput. Biol. *18*, e1010271. https://doi.org/10.1371/journal.pcbi.1010271.
24. Shan, S., Luo, S., Yang, Z., Hong, J., Su, Y., Ding, F., Fu, L., Li, C., Chen, P., Ma, J., et al. (2022). Deep learning guided optimization of human antibody against SARS-CoV-2 variants with broad neutralization. Proc. Natl. Acad. Sci. USA *119*, e2122954119. https://doi.org/10.1073/pnas.2122954119.
25. Lin, Z., Akin, H., Rao, R., Hie, B., Zhu, Z., Lu, W., Smetanin, N., Verkuil, R., Kabeli, O., Shmueli, Y., et al. (2023). Evolutionary-scale prediction of atomic-level protein structure with a language model. Science *379*, 1123–1130. https://doi.org/10.1126/science.ade2574.
26. Liberis, E., Veličković, P., Sormanni, P., Vendruscolo, M., and Liò, P. (2018). Parapred: antibody paratope prediction using convolutional and recurrent neural networks. Bioinformatics *34*, 2944–2950. https://doi.org/10.1093/bioinformatics/bty305.
27. Ruffolo, J.A., Gray, J.J., and Sulam, J. (2021). Deciphering antibody affinity maturation with language models and weakly supervised learning. Preprint at arXiv. https://doi.org/10.48550/arXiv.2112.07782.
28. Olsen, T.H., Moal, I.H., and Deane, C.M. (2022). AbLang: an antibody language model for completing antibody sequences. Bioinform. Adv. *2*, vbac046. https://doi.org/10.1093/bioadv/vbac046.
29. Olsen, T.H., Moal, I.H., and Deane, C.M. (2024). Addressing the antibody germline bias and its effect on language models for improved antibody design. Bioinformatics *40*, btae618. https://doi.org/10.1093/bioinformatics/btae618.
30. Kenlay, H., Dreyer, F.A., Kovaltsuk, A., Miketa, D., Pires, D., and Deane, C.M. (2024). Large scale paired antibody language models. PLoS Comput. Biol. *20*, e1012646. https://doi.org/10.1371/journal.pcbi.1012646.
31. Burbach, S.M., and Briney, B. (2024). Improving antibody language models with native pairing. Patterns *5*, 100967. https://doi.org/10.1016/j.patter.2024.100967.
32. Bepler, T., and Berger, B. (2021). Learning the Protein Language: Evolution, Structure and Function. Cell Syst. *12*, 654–669.e3. https://doi.org/10.1016/j.cels.2021.05.017.
33. Dunbar, J., Krawczyk, K., Leem, J., Baker, T., Fuchs, A., Georges, G., Shi, J., and Deane, C.M. (2014). SAbDab: the structural antibody database. Nucleic Acids Res. *42*, D1140–D1146. https://doi.org/10.1093/nar/gkt1043.
34. Schneider, C., Raybould, M.I.J., and Deane, C.M. (2022). SAbDab in the age of biotherapeutics: updates including SAbDab-nano, the nanobody structure tracker. Nucleic Acids Res. *50*, D1368–D1372. https://doi.org/10.1093/nar/gkab1050.
35. Punta, M., Coggill, P.C., Eberhardt, R.Y., Mistry, J., Tate, J., Boursnell, C., Pang, N., Forslund, K., Ceric, G., Clements, J., et al. (2012). The Pfam protein families database. Nucleic Acids Res. *40*, D290–D301. https://doi.org/10.1093/nar/gkr1065.
36. Raybould, M.I.J., Kovaltsuk, A., Marks, C., and Deane, C.M. (2021). CoV-AbDab: the Coronavirus Antibody Database. Bioinformatics *37*, 734–735. https://doi.org/10.1093/bioinformatics/btaa739.
37. Cao, Y., Jian, F., Wang, J., Yu, Y., Song, W., Yisimayi, A., Wang, J., An, R., Chen, X., Zhang, N., et al. (2023). Imprinted SARS-CoV-2 humoral immunity induces convergent Omicron RBD evolution. Nature *614*, 521–529. https://doi.org/10.1038/s41586-022-05644-7.
38. Cao, Y., Yisimayi, A., Jian, F., Song, W., Xiao, T., Wang, L., Du, S., Wang, J., Li, Q., Chen, X., et al. (2022). BA.2.12.1, BA.4 and BA.5 escape antibodies elicited by Omicron infection. Nature *608*, 593–602. https://doi.org/10.1038/s41586-022-04980-y.
39. Hu, E.J., Shen, Y., Wallis, P., Allen-Zhu, Z., Li, Y., Wang, S., Wang, L., and Chen, W. (2021). LoRA: Low-Rank Adaptation of Large Language Models. Preprint at arXiv. https://doi.org/10.48550/arXiv.2106.09685.
40. Oord, A. van den, Li, Y., and Vinyals, O. (2018). Representation Learning with Contrastive Predictive Coding. Preprint at arXiv. https://doi.org/10.48550/arXiv.1807.03748.
41. Khosla, P., Teterwak, P., Wang, C., Sarna, A., Tian, Y., Isola, P., Maschinot, A., Liu, C., and Krishnan, D. (2020). Supervised Contrastive Learning. Preprint at arXiv. https://doi.org/10.48550/arXiv.2004.11362.
42. Maaten, L. van der, and Hinton, G. (2008). Visualizing Data using t-SNE. J. Mach. Learn. Res. *9*, 2579–2605.
43. Lloyd, S. (1982). Least squares quantization in PCM. IEEE Trans. Inf. Theor. *28*, 129–137. https://doi.org/10.1109/TIT.1982.1056489.
44. Deshpande, A., Harris, B.D., Martinez-Sobrido, L., Kobie, J.J., and Walter, M.R. (2021). Epitope Classification and RBD Binding Properties of Neutralizing Antibodies Against SARS-CoV-2 Variants of Concern. Front. Immunol. *12*, 691715. https://doi.org/10.3389/fimmu.2021.691715.
45. Sankhala, R.S., Dussupt, V., Chen, W.-H., Bai, H., Martinez, E.J., Jensen, J.L., Rees, P.A., Hajduczki, A., Chang, W.C., Choe, M., et al. (2024). Antibody targeting of conserved sites of vulnerability on the SARS-CoV-2 spike receptor-binding domain. Structure *32*, 131–147.e7. https://doi.org/10.1016/j.str.2023.11.015.
46. Berman, H., Henrick, K., and Nakamura, H. (2003). Announcing the worldwide Protein Data Bank. Nat. Struct. Biol. *10*, 980. https://doi.org/10.1038/nsb1203-980.
47. Berman, H., Henrick, K., Nakamura, H., and Markley, J.L. (2007). The worldwide Protein Data Bank (wwPDB): ensuring a single, uniform archive of PDB data. Nucleic Acids Res. *35*, D301–D303. https://doi.org/10.1093/nar/gkl971.
48. wwPDB Consortium (2019). Protein Data Bank: the single global archive for 3D macromolecular structure data. Nucleic Acids Res. *47*, D520–D528. https://doi.org/10.1093/nar/gky949.
49. Scheid, J.F., Mouquet, H., Ueberheide, B., Diskin, R., Klein, F., Oliveira, T.Y.K., Pietzsch, J., Fenyo, D., Abadir, A., Velinzon, K., et al. (2011). Sequence and Structural Convergence of Broad and Potent HIV Antibodies That Mimic CD4 Binding. Science *333*, 1633–1637. https://doi.org/10.1126/science.1207227.
50. Scharf, L., Scheid, J.F., Lee, J.H., West, A.P., Chen, C., Gao, H., Gnanapragasam, P.N.P., Mares, R., Seaman, M.S., Ward, A.B., et al. (2014). Antibody 8ANC195 Reveals a Site of Broad Vulnerability on the HIV-1 Envelope Spike. Cell Rep. *7*, 785–795. https://doi.org/10.1016/j.celrep.2014.04.001.
51. Scharf, L., Wang, H., Gao, H., Chen, S., McDowall, A.W., and Bjorkman, P.J. (2015). Broadly Neutralizing Antibody 8ANC195 Recognizes Closed and Open States of HIV-1 Env. Cell *162*, 1379–1390. https://doi.org/10.1016/j.cell.2015.08.035.
52. Griffith, S.A., and McCoy, L.E. (2021). To bnAb or Not to bnAb: Defining Broadly Neutralising Antibodies Against HIV-1. Front. Immunol. *12*, 708227. https://doi.org/10.3389/fimmu.2021.708227.
53. Setliff, I., Shiakolas, A.R., Pilewski, K.A., Murji, A.A., Mapengo, R.E., Janowska, K., Richardson, S., Oosthuysen, C., Raju, N., Ronsard, L., et al. (2019). High-Throughput Mapping of B Cell Receptor Sequences to Antigen Specificity. Cell *179*, 1636–1646.e15. https://doi.org/10.1016/j.cell.2019.11.003.
54. Walker, L.M., Shiakolas, A.R., Venkat, R., Liu, Z.A., Wall, S., Raju, N., Pilewski, K.A., Setliff, I., Murji, A.A., Gillespie, R., et al. (2022). High-Throughput B Cell Epitope Determination by Next-Generation Sequencing. Front. Immunol. *13*, 855772. https://doi.org/10.3389/fimmu.2022.855772.
55. Zhou, T., Georgiev, I., Wu, X., Yang, Z.-Y., Dai, K., Finzi, A., Kwon, Y.D., Scheid, J.F., Shi, W., Xu, L., et al. (2010). Structural basis for broad and potent neutralization of HIV-1 by antibody VRC01. Science *329*, 811–817. https://doi.org/10.1126/science.1192819.
56. Wang, H., Gristick, H.B., Scharf, L., West, A.P., Jr., Galimidi, R.P., Seaman, M.S., Freund, N.T., Nussenzweig, M.C., and Bjorkman, P.J. (2017). Asymmetric recognition of HIV-1 Envelope trimer by V1V2 loop-targeting antibodies. eLife *6*, e27389. https://doi.org/10.7554/eLife.27389.

CellPress
OPEN ACCESS

57. Jespers, L.S., Roberts, A., Mahler, S.M., Winter, G., and Hoogenboom, H.R. (1994). Guiding the selection of human antibodies from phage display repertoires to a single epitope of an antigen. Biotechnology *12*, 899–903. https://doi.org/10.1038/nbt0994-899.

58. Sundararajan, M., Taly, A., and Yan, Q. (2017). Axiomatic Attribution for Deep Networks. Preprint at arXiv. https://doi.org/10.48550/arXiv.1703.01365.

59. Zielezinski, A. (2025). pfam_scan [Computer software]. GitHub. https://github.com/aziele/pfam_scan.

60. Shrake, A., and Rupley, J.A. (1973). Environment and exposure to solvent of protein atoms. Lysozyme and insulin. J. Mol. Biol. *79*, 351–371. https://doi.org/10.1016/0022-2836(73)90011-9.

61. Kabsch, W., and Sander, C. (1983). Dictionary of protein secondary structure: Pattern recognition of hydrogen-bonded and geometrical features. Biopolymers *22*, 2577–2637. https://doi.org/10.1002/bip.360221211.

62. Touw, W.G., Baakman, C., Black, J., te Beek, T.A.H., Krieger, E., Joosten, R.P., and Vriend, G. (2015). A series of PDB-related databanks for everyday needs. Nucleic Acids Res. *43*, D364–D368. https://doi.org/10.1093/nar/gku1028.

63. Henikoff, S., and Henikoff, J.G. (1992). Amino acid substitution matrices from protein blocks. Proc. Natl. Acad. Sci. USA *89*, 10915–10919.

64. Needleman, S.B., and Wunsch, C.D. (1970). A general method applicable to the search for similarities in the amino acid sequence of two proteins. J. Mol. Biol. *48*, 443–453. https://doi.org/10.1016/0022-2836(70)90057-4.

65. Delano, W. (2002). Pymol: An open-source molecular graphics tool. CCP4 Newsletter Protein Crystallography, 82–92.

66. Wolf, T., Debut, L., Sanh, V., Chaumond, J., Delangue, C., Moi, A., Cistac, P., Rault, T., Louf, R., Funtowicz, M., et al. (2019). HuggingFace's Transformers: State-of-the-art Natural Language Processing. Preprint at arXiv. https://doi.org/10.48550/arXiv.1910.03771.

67. Liu, Y., Ott, M., Goyal, N., Du, J., Joshi, M., Chen, D., Levy, O., Lewis, M., Zettlemoyer, L., and Stoyanov, V. (2019). RoBERTa: A Robustly Optimized BERT Pretraining Approach. Preprint at arXiv. https://doi.org/10.48550/arXiv.1907.11692.

68. Xie, X. jbloomlab SARS2_RBD_Ab_escape_data Xie_XS. https://media.githubusercontent.com/media/jbloomlab/SARS2_RBD_Ab_escape_maps/refs/heads/main/processed_data/escape_data.csv.

69. Greaney, A.J., Starr, T.N., and Bloom, J.D. (2022). An antibody-escape estimator for mutations to the SARS-CoV-2 receptor-binding domain. Virus Evol. *8*, veac021. https://doi.org/10.1093/ve/veac021.

70. Virtanen, P., Gommers, R., Oliphant, T.E., Haberland, M., Reddy, T., Cournapeau, D., Burovski, E., Peterson, P., Weckesser, W., Bright, J., et al. (2020). SciPy 1.0: fundamental algorithms for scientific computing in Python. Nat. Methods *17*, 261–272. https://doi.org/10.1038/s41592-019-0686-2.

71. Wu, X., Yang, Z.-Y., Li, Y., Hogerkorp, C.-M., Schief, W.R., Seaman, M.S., Zhou, T., Schmidt, S.D., Wu, L., Xu, L., et al. (2010). Rational Design of Envelope Identifies Broadly Neutralizing Human Monoclonal Antibodies to HIV-1. Science *329*, 856–861. https://doi.org/10.1126/science.1187659.

72. Alamyar, E., Duroux, P., Lefranc, M.-P., and Giudicelli, V. (2012). IMGT(®) tools for the nucleotide analysis of immunoglobulin (IG) and T cell receptor (TR) V-(D)-J repertoires, polymorphisms, and IG mutations: IMGT/V-QUEST and IMGT/HighV-QUEST for NGS. Methods Mol. Biol. *882*, 569–604. https://doi.org/10.1007/978-1-61779-842-9_32.

73. Alamyar, E., Giudicelli, V., Li, S., Duroux, P., and Lefranc, M.P. (2012). IMGT/HighV-QUEST: The IMGT web portal for immunoglobulin (IG) or antibody and T cell receptor (TR) analysis from NGS high throughput and deep sequencing. Immunome Res. *8*, 26–40. https://doi.org/10.4172/1745-7580.1000056.

74. Li, S., Lefranc, M.-P., Miles, J.J., Alamyar, E., Giudicelli, V., Duroux, P., Freeman, J.D., Corbin, V.D.A., Scheerlinck, J.-P., Frohman, M.A., et al. (2013). IMGT/HighV QUEST paradigm for T cell receptor IMGT clonotype diversity and next generation repertoire immunoprofiling. Nat. Commun. *4*, 2333. https://doi.org/10.1038/ncomms3333.

75. Giudicelli, V., Duroux, P., Lavoie, A., Aouinti, S., Lefranc, M.-P., and Kossida, S. (2015). From IMGT-ONTOLOGY to IMGT/HighVQUEST for NGS Immunoglobulin (IG) and T cell Receptor (TR) Repertoires in Autoimmune and Infectious Diseases. Autoimmun Infec Dis *1*. https://doi.org/10.16966/2470-1025.103.

76. Stewart-Jones, G.B.E., Chuang, G.-Y., Xu, K., Zhou, T., Acharya, P., Tsybovsky, Y., Ou, L., Zhang, B., Fernandez-Rodriguez, B., Gilardi, V., et al. (2018). Structure-based design of a quadrivalent fusion glycoprotein vaccine for human parainfluenza virus types 1–4. Proc. Natl. Acad. Sci. USA *115*, 12265–12270. https://doi.org/10.1073/pnas.1811980115.

77. Georgiev, I.S., Joyce, M.G., Yang, Y., Sastry, M., Zhang, B., Baxa, U., Chen, R.E., Druz, A., Lees, C.R., Narpala, S., et al. (2015). Single-Chain Soluble BG505.SOSIP gp140 Trimers as Structural and Antigenic Mimics of Mature Closed HIV-1 Env. J. Virol. *89*, 5318–5329. https://doi.org/10.1128/JVI.03451-14.

78. Holt, C.M., Figshare, https://doi.org/10.6084/m9.figshare.29647952.

Patterns, Volume *7*

Supplemental information

Contrastive learning enables epitope overlap predictions for targeted antibody discovery

Clinton M. Holt, Alexis K. Janke, Parastoo Amlashi, Parker J. Jamieson, Toma M. Marinov, and Ivelin S. Georgiev

Patterns, Volume *7*

Supplemental information

Contrastive learning enables epitope overlap predictions for targeted antibody discovery

Clinton M. Holt, Alexis K. Janke, Parastoo Amlashi, Parker J. Jamieson, Toma M. Marinov, and Ivelin S. Georgiev

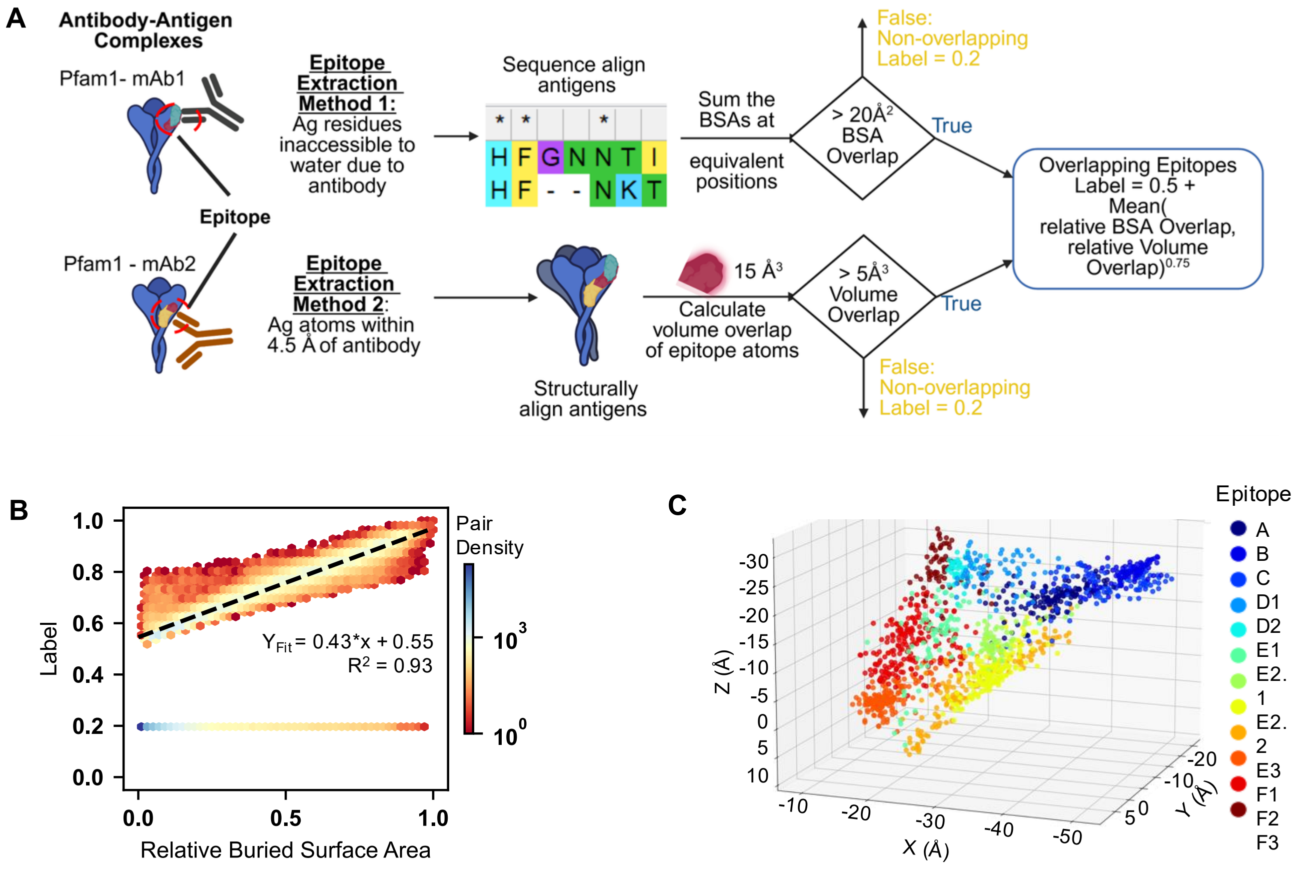

Figure S1. Quantitative Framework for Defining Epitope Relationships and Dataset Characteristics. (A) Approach for determining machine learning labels for antibody pairs targeting antigens within the same Pfam family. Two complementary metrics are employed. First, buried solvent accessible surface area (BSA) based residue-level epitope overlap, calculated as the solvent-accessible surface area buried by both antibodies at equivalent residues based on a pairwise sequence alignment. Antibody pairs where this value is less than 20 Å² are labeled as having non-overlapping epitopes. Second distance-based atom-level epitope overlap is calculated, defined by the volume overlap of antigen atoms within 4.5 Å of each antibody following antigen structural alignment (threshold >5 Å³). Finally, the epitope overlap is divided by the maximum possible epitope overlap to get relative overlaps. (B) Relationship between machine learning labels and relative buried surface area overlap visualized through hexagonal binning density plot. Only pairs with labels ≥ 0.5 were included for regression. Color intensity indicates pair density from minimum (red) to maximum (blue). (C) Three-dimensional visualization of deep mutational scanning (DMS) weighted average coordinates, with points colored by epitope classification. Coordinate calculation methodology is detailed in Methods, but briefly the antibody epitope is mapped onto the coordinates of SARS-CoV-2 RBD weighted by the extent each residue contributes to escape.

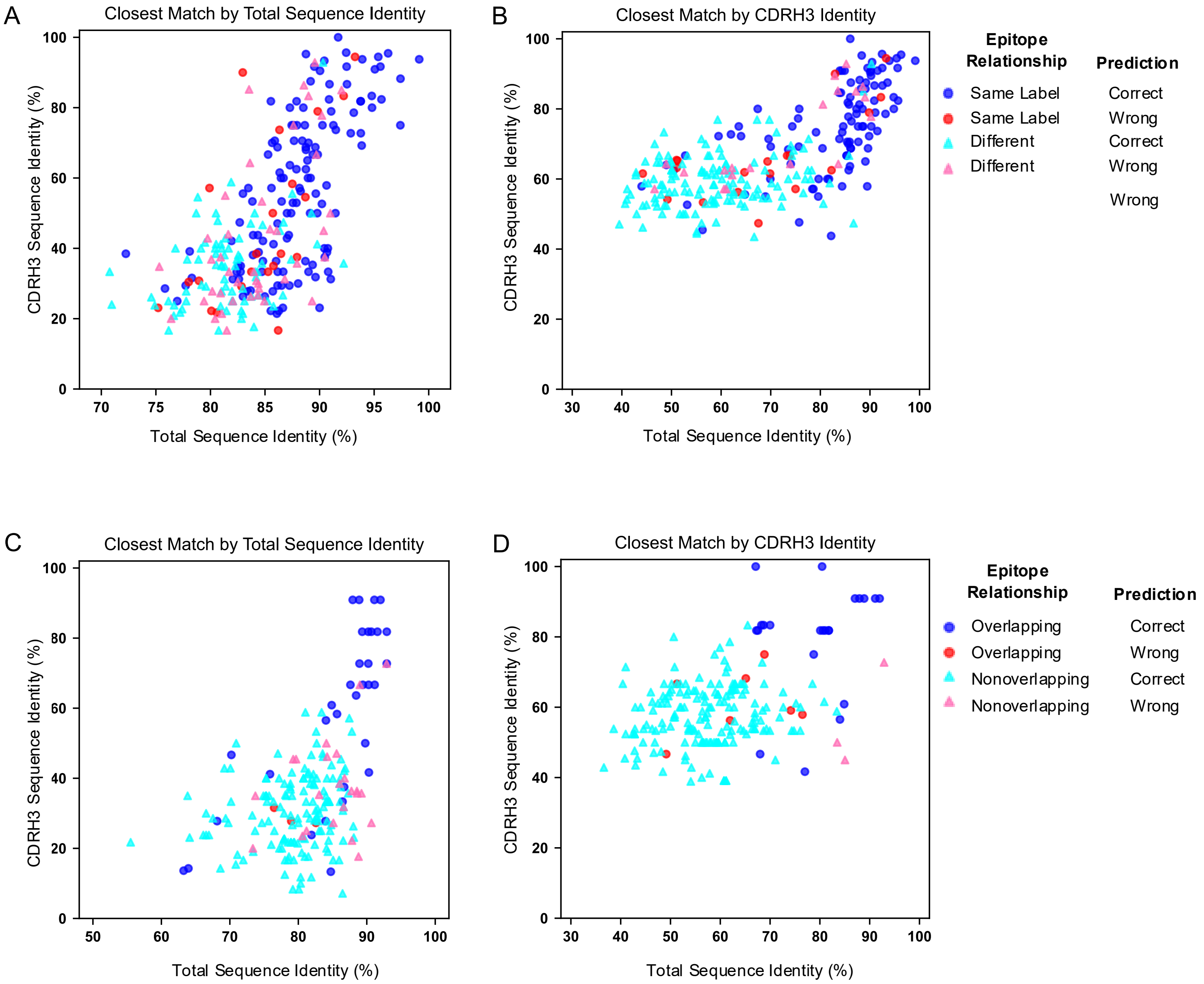

Figure S2: Test set antibodies have minimal sequence overlap with the training set. A-B) All SARS-CoV-2 RBD DMS test set antibodies are plotted as a single point representing how similar the most similar training set antibody is to it. The closest training set antibody is chosen by having the highest A) total percent amino acid sequence identity over both the heavy and light chains or B) CDRH3 percent amino acid sequence identity. C-D). The same analysis is repeated for the SAbDab test set antibodies. Pairings which have non-overlapping epitopes on the same pfam or bind different pfams are grouped together.

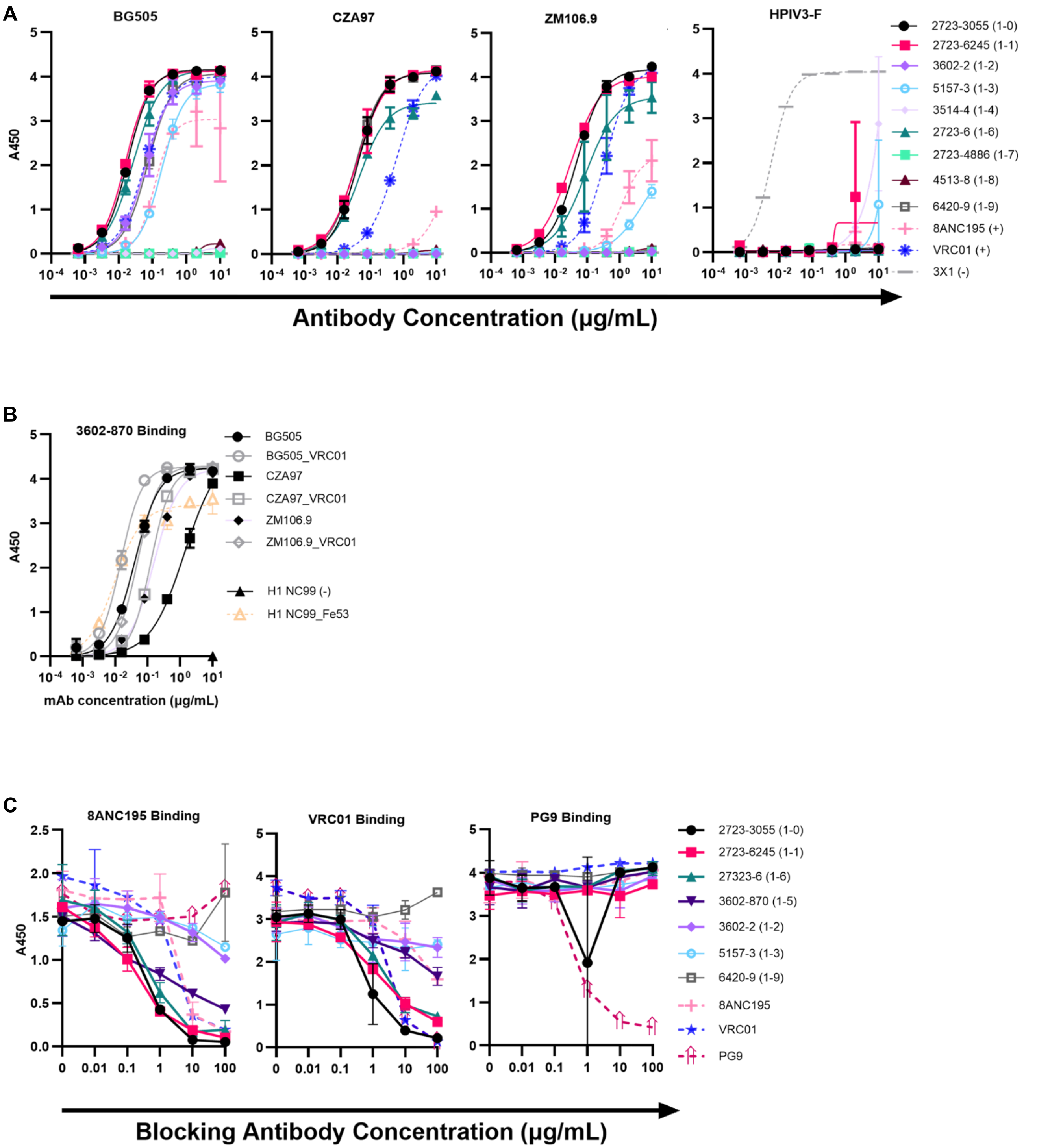

Figure S3: ELISA data for 8ANC195-targeted antibody discovery campaign. (A-B) ELISA curves pertaining to Figure 5C AUC values. Binding to trimeric HIV-1 envelope (BG505, CZA97, ZM106.9) or to a negative control antigen, human parainfluenza virus 3 fusion protein (HPIV3-F). (B) ELISA curves for antibody 3602-870, reproduced from data collected from 3602-870's initial publication. Antigens listed without underscores refer to the use of 3602-870 as the primary antibody whereas antibodies after underscores are positive controls for that antigen. H1 NC99 stands for the Influenza A hemagglutinin from the strain A/New Caledonia/20/99 (H1). (C) Competition ELISA curves showing detection of biotinylated antibody binding to BG505 SOSIP.664 with the biotinylated antibody as the title (8ANC195, VRC01, or PG9). Absorbance at 450 nm shown in presence of increasing concentrations of competitor antibodies. Data are represented as mean ± standard deviation. Filled symbols indicate mAbs displaying competition with 8ANC195.

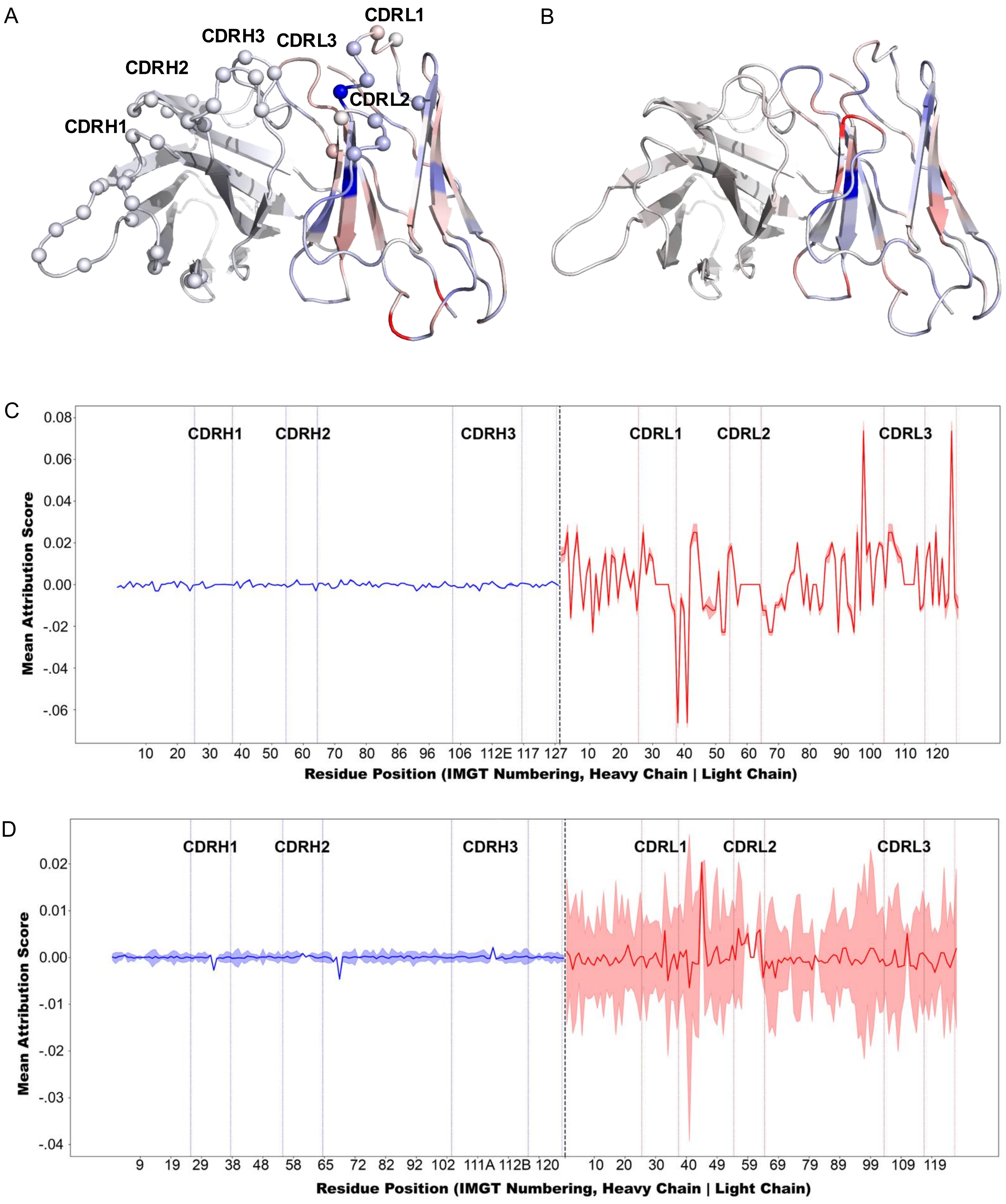

Figure S4. Feature Attribution Analysis Reveals the Light Chain Dominates Epitope Overlap Predictions. (A) Mean feature attributions for the 8ANC195 antibody (PDB ID 7KDE) versus five epitope-overlapping antibodies, mapped onto its structure. Complementarity-determining regions (CDRs) are labeled, and antigen-contacting residues (heavy atoms within 4.5 Å) are shown as spheres. (B) Mean feature attributions averaged over all test antibodies versus their nearest training neighbor, mapped onto the same structure for comparison. In both structures, colors scale from negative (blue) to positive (red) attribution. (C) The per-residue attribution scores used for panel A, plotted for the heavy (blue) and light (red) chains with CDRs highlighted. (D) The per-residue attribution scores used for panel B, averaged at each IMGT position across the test set; the shaded region represents one standard deviation.
